# A cytochrome P450 insecticide detoxification mechanism is not conserved across the Megachilidae family of bees

**DOI:** 10.1111/eva.13625

**Published:** 2023-12-06

**Authors:** Angela Hayward, Benjamin J. Hunt, Julian Haas, Ellie Bushnell‐Crowther, Bartlomiej J. Troczka, Adam Pym, Katherine Beadle, Jeremy Field, David R. Nelson, Ralf Nauen, Chris Bass

**Affiliations:** ^1^ Centre for Ecology and Conservation University of Exeter Penryn, Cornwall UK; ^2^ Bayer AG, Crop Science Division Monheim Germany; ^3^ Department of Molecular Sciences University of Tennessee Memphis Tennessee USA

**Keywords:** ecotoxicology, gene structure and function, molecular evolution

## Abstract

Recent work has demonstrated that many bee species have specific cytochrome P450 enzymes (P450s) that can efficiently detoxify certain insecticides. The presence of these P450s, belonging or closely related to the CYP9Q subfamily (CYP9Q‐related), is generally well conserved across the diversity of bees. However, the alfalfa leafcutter bee, *Megachile rotundata*, lacks CYP9Q‐related P450s and is 170–2500 times more sensitive to certain insecticides than bee pollinators with these P450s. The extent to which these findings apply to other Megachilidae bee species remains uncertain. To address this knowledge gap, we sequenced the transcriptomes of four *Megachile* species and leveraged the data obtained, in combination with publicly available genomic data, to investigate the evolution and function of P450s in the Megachilidae. Our analyses reveal that several Megachilidae species, belonging to the Lithurgini, Megachilini and Anthidini tribes, including all species of the *Megachile* genus investigated, lack *CYP9Q*‐related genes. In place of these genes *Megachile* species have evolved phylogenetically distinct *CYP9* genes, the *CYP9DM* lineage. Functional expression of these P450s from *M. rotundata* reveal they lack the capacity to metabolize the neonicotinoid insecticides thiacloprid and imidacloprid. In contrast, species from the Osmiini and Dioxyini tribes of Megachilidae have *CYP9Q*‐related P450s belonging to the *CYP9BU* subfamily that are able to detoxify thiacloprid. These findings provide new insight into the evolution of P450s that act as key determinants of insecticide sensitivity in bees and have important applied implications for pesticide risk assessment.

## 1 INTRODUCTION

Bees are the most important group of insect pollinators, playing a crucial economic and ecological role in ensuring human crop production and the stability of natural ecosystems (Ollerton, 2017; Woodcock et al., 2019). Indeed, up to 35% of global food production, depends on bee pollinators (Ollerton, 2017; Potts, 2016). Although pollination services can be provided by managed bee pollinators, such as the western honeybee (*Apis mellifera*) and alfalfa leafcutter bee (*Megachile rotundata*), the vast majority of bee pollinators are non‐managed, wild taxa (Ollerton, 2017; Pitts‐Singer & Cane, 2011; Woodcock et al., 2019). There is growing concern about the reported decline of insect pollinator species, and the resulting potential impacts on biodiversity and future food security (Biesmeijer et al., 2006; Leather, 2018; Seibold et al., 2019; Wagner, 2020). Pollinator declines have been reported in all continents, except Antarctica, and bees are not exempt from this trend (FAO, 2008). As an example, 9.2% of wild bee populations in Europe have been given the IUCN Red List status of ‘threatened’, and there is also good evidence for a global decline in species of bumblebee (*Bombus* species) (Cameron et al., 2011; Nieto et al., 2014; Williams & Osborne, 2009). The reasons for these declines are complex and are thought to include changing land‐use, pathogens and parasites, climate change, agricultural intensification and the use of insecticides (Carvell et al., 2006; Goulson et al., 2005, 2015; Nieto et al., 2014). *A. mellifera* is an important model for bee pollinators and the potential impact of insecticides on the health of this species has been the subject of numerous scientific publications (Hardstone & Scott, 2010; Johnson et al., 2010; Sanchez‐Bayo et al., 2016; Wood et al., 2020). It is also used as a proxy for other bee species in the assessment of insecticide toxicity on non‐target arthropods within existing regulatory requirements (EFSA, 2013). However, there is significant uncertainty over the extent to which data generated for *A. mellifera* can be applied to wild bee populations (Allen‐Wardell et al., 1998; Klein et al., 2007; Wood et al., 2020).

Bees (Anthophila) evolved from a carnivorous wasp ancestor, approximately 100–149 mya and, although monophyletic, they rapidly diversified into seven families (Hedtke et al., 2013; Litman et al., 2011; Michener, 2007). Given the large number of bee species (~20,000), it is not surprising that there is a wide variety in life‐history traits such as nesting biology, host‐plant associations and foraging behaviours, potentially exposing species to distinct repertoires of plant secondary metabolites (Armbruster, 2017; Danforth et al., 2019; Michener, 2007). In common with other insects, bees have evolved sophisticated detoxification systems that can either metabolize endogenous substrates and xenobiotics into non‐toxic compounds, or produce metabolites that can be eliminated rapidly (Panini et al., 2016). These detoxification pathways are vital in determining the level of sensitivity of bees to insecticides (Berenbaum & Johnson, 2015). In many taxa of bees, the P450 superfamily has been most frequently implicated in the metabolic detoxification of naturally occurring and synthetic xenobiotics (Iwasa et al., 2004; Johnson et al., 2006, 2012; Nauen et al., 2022). Indeed, recent work has shown that cytochrome P450 enzymes belonging to the CYP9Q‐subfamily, and its closely related lineages, found across all major bee families, are capable of metabolizing certain synthetic insecticides from at least four classes, including the *N*‐cyanoamidine neonicotinoid thiacloprid, the butenolide flupyradifurone, the pyrethroid *tau*‐fluvalinate and the organophosphate coumaphos (Beadle et al., 2019; Haas et al., 2022; Manjon et al., 2018; Mao et al., 2011; Reid et al., 2020). These findings reveal that P450 enzymes belonging to the CYP9Q‐related subfamily can function as potent generalist metabolizers, capable of binding multiple toxins and detoxifying them, in a similar way to human CYP3A4 and CYP2D6 (Haas et al., 2022; Manjon et al., 2018; Reid et al., 2020; Zanger & Schwab, 2013).

Although *CYP9Q*‐related genes appear to be ubiquitous across most bee families, recent work has shown that their presence is not universal in the Megachilidae (Haas et al., 2022; Hayward et al., 2019). The Megachilidae is a large, cosmopolitan family, containing over 4000 species of bee, with taxa found on all continents, with the exception of Antarctica (Gonzalez et al., 2012; Michener, 2007). Some Megachilidae species, such as *Osmia bicornis*, have *CYP9BU* genes, which are closely related to *CYP9Q* sequences and have a similar capacity to metabolize certain insecticide chemotypes (Beadle et al., 2019). In contrast, other Megachilidae appear to have lost the *CYP9BU* lineage entirely or, as in the case of *M. rotundata*, have evolved a divergent lineage of CYP9s that are not closely related to CYP9Q P450s (Haas et al., 2022; Hayward et al., 2019). Importantly, the absence of CYP9Q‐type P450s in this species was correlated with high sensitivity to insecticides that are classified as moderately toxic or practically non‐toxic, by the US Environmental Protection Agency, in bees with these P450s (USEPA et al., 2014). For example in acute contact bioassays *M. rotundata* was found to be up to 2500‐fold more sensitive to the *N*‐cyanoamidine neonicotinoid thiacloprid and 170‐fold more sensitive to the butenolide flupyradifurone than *A. mellifera* (Hayward et al., 2019). This finding has major implications for the safe use of insecticides in crops where *M. rotundata* is used for pollination (Haas et al., 2022; Hayward et al., 2019). Unfortunately, genomic data for the Megachilidae remains limited and thus the extent to which these findings apply to other bees of the Megachile genus remains uncertain. Furthermore, the capacity of non‐CYP9Q‐type P450 subfamilies to metabolize insecticides remains to be functionally established.

The aim of the current study was to provide insight into this knowledge gap by addressing the following key questions: (1) How widespread is the loss, or evolutionary substitution, of this important subfamily of P450s within the Megachilidae? (2) Do all CYP9BU P450s have the conserved capacity to detoxify insecticide observed in the other *CYP9Q‐*related lineages? (3) Does our current use of *A. mellifera* as a proxy in toxicology testing afford adequate protection to species in the Megachilidae?

## 2 METHODS

### 2.1 Chemicals

The coumarin fluorescent model substrate 7‐(4‐methoxybenzyloxy)‐4‐trifluoromethyl coumarin (MOBFC) was obtained as analytical grade from Invitrogen. All remaining chemicals were of the highest purity available and obtained from Sigma‐Aldrich (St. Louis, USA).

### 2.2 Curation of the CYPome of four Megachile species

A male bee of a native Canadian species emerged alongside commercially supplied *M. rotundata* (13.08.18) (obtained from Canada through Bayer AG Crop Science Division). Using the Canadian Journal of Arthropod Identification No. 18, available online (https://cjai.biologicalsurvey.ca/srpg_18/C39/C39.html) it was identified as *M. lapponica* Thomson. Three UK native *Megachile* species were collected in July 2019. Female *M. centuncularis* (L.) and *M. willughbiella* (Kirby) were collected from Falmouth Cornwall (50°8′48.69″ N, 5°3′51.20″ W) and female *M. leachella* Curtis were collected at Porthleven, Cornwall (50°4′9.87″ N, 5°17′39.83″ W). All specimens were snap‐frozen in liquid N_2_ and stored at −80°C.

RNA was extracted using the Bioline Isolate II RNA Mini Kit (Bioline Reagents) following the manufacturers recommended protocol. RNA was eluted in 30 μL RNase‐free water and stored at −80°C. The quality and quantity of the RNA was assessed using Qubit® RNA BR and Qubit® RNA IQ Assay kits, and RNA integrity was visualized on an agarose gel.

Transcriptome libraries (250–300 bp insert cDNA) were generated by Novogene UK (Cambridge, CB4 0FW, UK; https://en.novogene.com/) from 10,000 ng total RNA, and run using high‐throughput sequencing (Illumina HiSeq) with 150 bp paired‐end chemistry, with 30 Gb raw data obtained per sample. Quality control was run by Novogene using an Agilent 2100. The qualified libraries were pooled according to concentration and expected data volume and then run on an Illumina sequencer.

For each dataset, the reads were quality trimmed, in silico normalized and assembled using Trinity version 2.8.4 (Grabherr et al., 2011). Trimming was performed using Trimmomatic (Bolger et al., 2014) as implemented in Trinity with default settings, and strand‐specific assembly was specified using the parameter—SS_lib_type RF. Assembly statistics were generated using TransRate v1.0.3 (https://genome.cshlp.org/content/26/8/1134). Peptide sequences were identified using TransDecoder v5.5.0 (https://github.com/TransDecoder/TransDecoder), clustered at 95% identity using CD‐HIT (https://academic.oup.com/bioinformatics/article/28/23/3150/192160) to reduce redundancy, and used to assess transcriptome completeness using the BUSCO Arthropoda gene set (https://academic.oup.com/bioinformatics/article/31/19/3210/211866).

The resulting contigs were imported, in fasta file format, into Geneious version 10.2.3 (Biomatters) and a BLAST database was created from each dataset. The nucleotide sequences for *A. mellifera CYP9Q3*, *CYP9P1*, *CYP9R1*, *M. rotundata CYP9DN1* and *CYP9DM1* were used as the query sequences in a discontiguous BLAST search through the database for each species. The resulting nucleotide sequences were translated and manually inspected for the presence of conserved motifs. Partial sequences and duplicates were removed. The *Megachile* CYP9 sequences were then named using the recognized P450 nomenclature (Nelson et al., 1993, 1996).

### 2.3 Phylogenetic analyses of the CYP9 subfamily across bee families

The NCBI genome database (https://www.ncbi.nlm.nih.gov/genome/) was searched for published genomes of ‘Apoidea (bees); taxid: 34735’. The nucleotide sequences for *A. mellifera CYP9Q3* (XM_006562300.3), *CYP9P1* (XM_006562302.3) and *CYP9R1* (XM_026445177.1) were used as the query sequences in a BLASTN search through the assembly of the genome of each bee species to find CYP9 homologs. A TBLASTN translated protein similarity search was also performed using the same query sequences. Scaffolds containing CYP9 P450s were imported into Geneious version 10.2.6 (Biomatters). The nucleotide sequences for *A. mellifera CYP9Q3*, *CYP9P1*, and *CYP9R1* were used as the query sequences in a BLASTN search through the TSA database, limited by bee species to find CYP9 homologs. CYP9 sequences for each bee species were translated and inspected for the presence of conserved motifs. Partial sequences were removed. CYP9 sequences were named numerically for each species (e.g. *Lasioglossum albipes*: La_CYP9‐like1, La_CYP9‐like2).

The CYP9 protein sequences were aligned with the outgroup *Nasonia vitripennis* CYP9AG4 (NP_001166010.1) in Geneious using MUSCLE (Edgar, 2004). MEGAX (Kumar et al., 2018) was used to find the best‐fit model of amino acid substitution, using a maximum likelihood fit of 56 different models. The substitution model with the lowest Bayesian Information Criterion (BIC) score was selected for use in phylogeny estimation. Phylogeny was generated using Bayesian inference (Huelsenbeck & Ronquist, 2001) [Substitution model: LG + G (Le & Gascuel, 2008); Chain length: 1,100,000; Subsampling frequency: 200; Burn‐in length: 100,000; Heated chains: 4; Heated chain temperature: 0.2].

### 2.4 Syntenic analyses of the CYP9 cluster across bee families

Genomic sequences containing CYP9 sequences were retrieved from the NCBI database for: *A. mellifera* (DH4 linkage group LG14, Amel_HAv3.1 WGS), *B. terrestris* (LG B01, Bter_1.0 WGS), *M. rotundata* (MROT_1.0: scf_0244), *O. bicornis* (Obicornis_v3: scf00060), *A. xeuzhongi* (CM039649: chromosome 14), *D. novaeangliae* (ASM127255v1: scaffold21), *C. gigas* (ASM1312311v1: WUUM01000008), *A. haemorrhoa* (OU342941: chromosome 2) and *M. europaea* (OU744344: chromosome 2). Syntenic analysis between these scaffolds (macro‐synteny) was performed using Mauve (multiple alignment of conserved genomic sequence with rearrangements) v2.4.0 (Darling et al., 2004, 2010). This allowed the order and orientation of segments to be displayed and all locally collinear blocks (LCBs) to be defined.

The region ~200 kbp upstream and downstream of the CYP9 genes was examined for micro‐synteny using Mauve v2.4.0 (Darling et al., 2004, 2010), using *A. mellifera* as the reference. The minimum requirement for micro‐synteny was the conservation of two neighbouring homologs with no more than five unrelated genes in the intervening DNA.

### 2.5 Functional analyses of CYP9 sequences from the Megachilidae family

The Megachilidae P450 genes were synthesized by TWIST biosciences and delivered in the pENTR entry plasmid. Expression clones were created through Gateway® cloning using BaculoDirect™ baculovirus expression system (Invitrogen) according to the manufacturer's instructions. Functional expression of recombinant P450 proteins was conducted in high‐five (*Trichoplusia ni*) insect cells, co‐infected with house‐fly (*Musca domestica*) NADPH‐dependent cytochrome P450 reductase (CPR) as recently described (Haas et al., 2021; Manjon et al., 2018). Cells were harvested after 60 h, centrifuged and the cell pellet stored at −80°C until microsomal membrane preparation was performed according to standard protocols (Janmohamed et al., 2006) with minor changes. In brief, the cell pellets were homogenized in ice‐cold 0.1 M potassium phosphate buffer (pH 7.6) containing 1 mM EDTA, 1 mM DTT and 200 mM saccharose using a sonifier (Branson digital sonifier 250) at 50% amplitude, pulsing on for 2 s, off for 2 s, for 60 s and centrifuged at 2700 rpm at 4°C for 10 min. The supernatant was centrifuged at 4°C at 100,000*g* for 1 h, and the pellet resuspended in 0.1 M potassium phosphate buffer (pH 7.6) containing 0.1 mM EDTA, 1 mM DTT and 10% glycerol using a glass homogenizer. A CO‐difference spectral assay, adapted from Guengerich et al. (2009), was performed for each recombinant P450 to estimate expression and function levels, using a Specord 200 Plus Spectrophotometer (Analytik Jena). The protein content of samples was established using a Bradford's protein assay and bovine serum albumin as a reference standard.

#### 2.5.1 Fluorescence‐based model substrate profiling

The activity of the recombinant P450 proteins was screened against a panel of six fluorescent coumarin model substrates: 7‐ethoxy‐coumarin (EC), 7‐methoxy‐coumarin (MC), 7‐ethoxy‐4‐(trifluoromethyl)‐coumarin (EFC), 7‐benzyloxy‐4‐(trifluoromethyl)‐coumarin (BFC), 7‐methoxy‐4‐(trifluoromethyl)‐coumarin (MFC) and 7‐(4‐methoxybenzyloxy)‐4‐trifluoromethyl‐coumarin (MOBFC). Assays were performed in a black flat‐bottomed 96‐well plate (4‐titude) with 100 μL total reaction and three technical replicates for each data point. Reactions contained 50 μg protein per well, 1 mM of NADPH and 50 μM coumarin model substrate. P450 proteins were incubated without NADPH and wells containing only potassium phosphate buffer served as controls. Samples were incubated at 25 ± 1°C for 1 h and data were recorded using a SpectraMax M2 multi‐mode plate reader (Molecular Devices) at the excitation/emission wavelength suitable for each model substrate (EC, MC at 390/465; EFC at 410/510; BFC, MFC at 410/535 and MOBFC at 405/510 nm). EC and MC have a similar emission wavelength (465 nm) to NADPH (460 nm) and so reactions for these substrates were terminated after the 1 h incubation prior to measurement by the addition of 100 μL of a stop solution (25% DMSO, 0.05 M Tris/HCL pH 10, 5 mM glutathione oxidized and 0.2 U glutathione reductase). These plates were incubated at 25 ± 1°C for a further 50 min before data was recorded. HC was used as a reference for the smaller coumarins, EC and MC, and HFC as a reference for the larger coumarins, EFC, BFC, MFC and MOBFC, in order to generate standard curves as previously described (Manjon et al., 2018).

#### 2.5.2 LC–MS/MS analyses of neonicotinoid metabolism

Recombinant P450s (160 μg/well) in 0.1 M potassium phosphate buffer were incubated for 1 h (shaking at 225 rpm) with neonicotinoid insecticides, at a concentration of 10 μM, in a total assay volume of 200 μL, at 30 ± 1°C, in the presence or absence of a NADPH regeneration system. Three replicates were performed for each data point. Samples incubated without NADPH served as controls. The reactions were terminated by the addition of ice‐cold acetonitrile (to 80% final concentration). Samples were incubated at 4°C overnight and then centrifuged at 3000*g* for 10 min to pellet any precipitation of protein. LC–MS/MS was performed as previously described (Haas et al., 2022), using an Acquity UPLC (Waters) coupled to an API 4000 mass spectrometer (Sciex) and an Infinity II UHPLC (Agilent Technologies; reverse phase mode) coupled to a QTRAP 6500 mass spectrometer (Sciex) employing electrospray ionization. The recovery rates of parent compounds incubated without NADPH were typically close to 100%.

### 2.6 Statistical analyses

All statistical analyses were performed in GraphPad Prism 7 (GraphPad Software). Significant differences in the activity of recombinant P450s against thiacloprid and imidacloprid were determined using a Welch's *t*‐test. The level of the known metabolites hydroxy‐thiacloprid (TCP‐OH) and hydroxy‐imidacloprid (IMI‐OH) in the controls for all the P450s tested was below the limit of quantification (<LOQ), and so metabolite production was compared across the two lineages of enzymes (CYP9BU and CYP9DM) using an ANOVA and *post‐hoc* Dunnett's multiple comparison tests. Statistical details of experiments (value of *n*, precision measures and definitions of significance) are provided in figure legends.

### 2.7 Modelling of bee CYP9 P450 proteins

Three‐dimensional models of *A. mellifera* CYP9Q3, *M. rotundata* CYP9DM1 and *C. florisomne* CYP9BU3 were generated using the AlphaFold2 structure software (Jumper et al., 2021; Mirdita et al., 2022). The primary sequences of the three CYP9 P450s were submitted to the ColabFold web interface (Mirdita et al., 2022) and the default options were chosen to run the software, with the multiple sequence alignment step performed using MMseqs2 (Mirdita et al., 2019). The stereochemical soundness and quality of the predicted models were assessed using the Structural Analysis and Verification Server (https://saves.mbi.ucla.edu/), and ERRAT scores (Colovos & Yeates, 1993), PROCHECK data (Laskowski et al., 1996) and Ramachandran plots were generated.

To predict the position of the heme molecule, the AlphaFill database was searched to provide the closest available match to the models predicted in this study (Hekkelman et al., 2023). Two species of bee, *Apis cerana cerana* (Oriental honeybee) and *O. bicornis* (Red mason bee, previously known as *Osmia rufa*), had CYP9 P450 sequences available with UniProt entry IDs. *A. cerana cerana* CYP9Q3 (Entry ID: A0A1D6XRL0) and *O. bicorns* CYP9BU1 (Entry ID: A0A411K6V7) were used to search the AlphaFill database (https://alphafill.eu) for an AlphaFold2 predicted model with associated ligands and co‐factors. The AlphaFold models generated for this project were aligned with both AlphaFill models using Schrödinger Maestro Suite v13.3 (Schrödinger, USA), and alignment and RMSD scores were calculated. The AlphaFill model that generated the lowest alignment score was selected to determine the position of the heme molecule. *A. cerana cerana* CYP9Q3 was used with *A. mellifera* CYP9Q3 (Alignment score: 0.028), and *O. bicornis* CYP9BU1 was used with *C. florisomne* CYP9BU3 and *M. rotundata* CYP9DM1 (Alignment scores: 0.052 and 0.202 respectively). The models were refined for further analysis by adding hydrogens, creating zero‐order bonds to metals and disulphide bonds, and the heme iron (Fe) charge set to +3. The highly conserved cysteine residue was examined for the presence of a hydrogen bond (salt bridge) to Fe^3+^ in the heme molecule.

These prepared models were exported as ‘holoenzymes’ in a .pdb format and uploaded to Caver Web 1.1 for further analysis (Stourac et al., 2019). The volume (Å^3^), relevance score and druggability score of the catalytic pockets were estimated for each model, before access tunnel detection was conducted. Druggability score is used to categorize proteins into four classes (≥1.0: very druggable, 0.8–1.0: druggable, 0.7–0.8: intermediate and ≤0.7: difficult). Subsequent transport analyses were conducted for the two neonicotinoid insecticides, thiacloprid and imidacloprid, for all relevant access tunnels detected.

The holoenzymes were exported to UCSF Chimera v 1.16 and the secondary structure was examined with reference to the structures known to be implicated in binding and function in other P450s. In particular the F/G and B/C helix‐rich regions and the I‐helix were identified. The residues identified as making up the catalytic pockets by Caver Web 1.1 were also identified on the structures. The surface of the protein was visualized and then the electrostatic potential was calculated according to Coulomb's law: *ϕ* = ∑[*q*_*i*_/(*εd*_*i*_)], where *ϕ* is the potential, *q* are the atomic charges, *d* are the distances from the atoms and *ε* is the dielectric, using the default settings (*ε* = 4*r*, distance from surface = 1.4 Å, thresholds ±10 kcal/mol*e).

## 3 RESULTS

### 3.1 Curation of the CYPomes of four Megachile species

As detailed in the introduction, our recent work has revealed the loss of a P450 subfamily that acts as a key determinant of insecticide sensitivity in some Megachilidae species (i.e. members of the *Megachile* genus). However, genomic resources for individual *Megachile* species are severely limited, with data only available for two species in our previous analysis. To address this, we first sought to extend data of the CYPome of *Megachile* species by sequencing the transcriptome of additional *Megachile* species. RNA extracted from one Canadian (*M. lapponica*) and three UK species of *Megachile* (*M. centuncularis*, *M. leachella* and *M. willughbiella*) were sequenced. All the RNAseq samples yielded a high number and percentage of clean reads (2.14 × 10^8^–2.99 × 10^8^; 96.9%–98.77%). These were de novo assembled into a total of 185,210, 241,588, 148,465 and 204,567 transcripts in the case of *M. centuncularis*, *M. leachella*, *M. lapponica* and *M. willughbiella* respectively with mean contig lengths for each species ranging from 1782 to 2505 (Table S1). Assessment using the BUSCO Arthropoda gene set indicated >99% completeness for all assemblies (Table S1). The assemblies were mined for P450 sequences, and the full repertoire of P450 sequences identified in each of the *Megachile* bees is shown in Table S2. The CYP9 subfamily in these species is broadly similar to that observed in *M. rotundata* (Tables S2 and S3), with all four *Megachile* species having at least one *CYP9DM* gene. *M. willughbiella* has undergone an expansion of this lineage and has three *CYP9DM* genes (*CYP9DM5*, *CYP9DM6* and *CYP9DM7*). None of the *Megachile* species has a sequence that is highly similar to a *CYP9Q*‐related gene.

### 3.2 Phylogenetic analyses of the CYP9 subfamily across six bee families reveals a unique gene lineage in the Megachile genus

**FIGURE 1:**
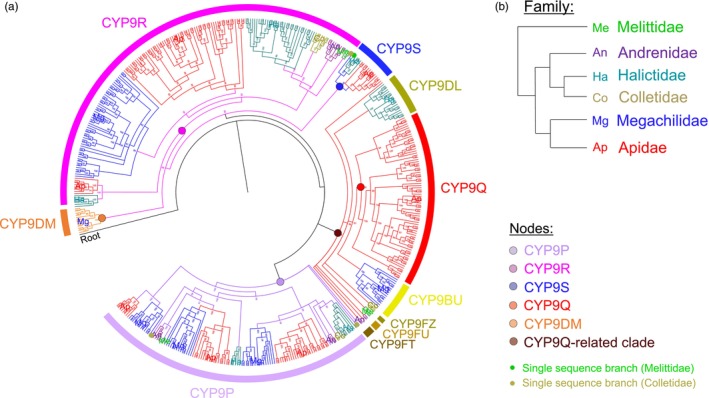
Phylogeny of the CYP9 subfamily of P450s across 81 bee species, including five Megachile species (*M. centuncularis*, *M. lapponica*, *M. leachella*, *M. rotundata* and *M. willughbiella*). (a) Phylogenetic tree of CYP9 P450s estimated using Bayesian inference. Node support is shown as % posterior probability. Tree is rooted on *Nasonia vitripennis* CYP9AG4 (NCBI reference sequence: NP_001166010.1). CYP9 sequences cluster to form distinct lineages: CYP9P (purple), CYP9R (pink), CYP9S (blue), CYP9Q‐related [further divided by family into: CYP9Q (red), CYP9BU (yellow), CYP9DL (gold), CYP9FZ (dark gold), CYP9FU (light brown) and CYP9FT (dark brown) sequences] and the Megachile‐specific CYP9DM (orange). Outer leaves of CYP9 lineages are coloured by bee family and annotated with an abbreviated form of the family name. Single sequence branches are labelled with a circle, coloured by family. With the exception of the sequences from *M. centuncularis*, *M. lapponica*, *M. leachella* and *M. willughbiella*, which were obtained from RNAseq data, all sequences used were accessed from the NCBI databases. (b) Schematic of the phylogenetic relationship between bee families.

The transcriptomic data for the four species of *Megachile* sequenced were combined with the sequences retrieved from the public databases to examine the evolutionary relationship of the CYP9 subfamily across six bee families in greater depth, using phylogenetic analyses (Figure 1). No genomic data was available for the Stenotritidae, which is comprised of two genera with a complement of just 21 species, all of which are endemic to Australia. The major bee families were represented by 4 Melittidae, 3 Colletidae, 10 Halictidae, 6 Andrenidae, 17 Megachilidae and 41 Apidae species. In all, 562 CYP9 sequences were identified. The *CYP9DN* gene is located on a separate chromosome (scaffold) in the genome of bees and is therefore not part of the main CYP9 locus, these were removed before analysis, leaving a total of 424 sequences. The majority of the nodes in the phylogeny had strong Bayesian posterior probability support (>80%). Although the support values dropped for some of the deeper nodes, where more uncertainty is expected, none were lower than 50%.

In agreement with Haas et al. (2022), the topology of this phylogeny revealed that the *CYP9Q*‐related lineage (*CYP9BU*s) are not universal within the Megachilidae, with fewer than 38% of the species having at least one *CYP9BU*. The six Megachilidae species with CYP9BU sequences detected in the phylogenetic analyses were: three *Osmia* species (*O. bicornis*, *O. cornuta and O. lignaria* tribe: Osmiini), *Heriades truncorum* (tribe: Osmiini), *Chelostoma florisomne* (tribe: Osmiini) and *Dioxys cincta* (tribe: Dioxyini) (Figure 1; Figure S1). Those species with an apparent loss are members of the Megachilini (five species), Anthidiini (three species) and Lithurgini (one species) tribes (Figure 1; Figure S1). In contrast, the presence of *CYP9Q*‐related genes in the other bee families was ubiquitous, with over 92% of species having at least one full‐length sequence, a figure that rose to 100% when partial sequences from the TSA database were considered (Figure 1). Overall, this strongly implies a loss of this important gene family in certain Megachilidae species.

Of the 81 species included in the phylogenetic analyses only members of the *Megachile* genus have *CYP9DM* genes (Figure 1). This gene lineage clusters in association with the *CYP9R* and *CYP9S* genes and is phylogenetically distant from the *CYP9Q*‐related lineage. The root branch lengths for the *CYP9P*, *CYP9Q*, *CYP9R* and *CYP9S* lineages are 0.17, 0.23, 0.09 and 0.19 substitutions per site, respectively (Figure 1). Whereas the *CYP9DM* lineage has the longest branch length in the phylogeny, with a root branch of 0.72 substitutions per site, which is indicative of an increased level of genetic change in this gene line. Four of the five conserved motifs common to P450s show a high level of consensus across the CYP9 sequences included in the phylogeny (Dataset S1). Only the second motif, the oxygen‐binding domain, Gx[E/D][T/S][IV], of the *CYP9DM* genes shows divergence. Whereas almost all the other CYP9 sequences (>99.5%, *n* = 413) have the canonical negatively charged residue (E/D) in the third position of the motif, the *CYP9DM* sequences (*n* = 9) have a substitution for either alanine (A) or threonine (T) (Dataset S1). Other than the *Megachile CYP9DM* sequences only two CYP9 P450s have a substitution of the canonical charged residue. *Nomia melanderi* (Halictidae) and *Eucera syriaca* (Apidae) have a substitution to asparagine (N) and histidine (H), respectively. However, both of these have the conserved G at the start of the motif (Dataset S1). Taken together, the apparent loss of a gene lineage in certain members of the Megachilidae and the evolution of at least one genus‐specific lineage are suggestive of divergent selection acting on the CYPome of some members of this cosmopolitan bee family.

### 3.3 Analyses of gene synteny confirms the conserved genomic landscape of the CYP9 cluster across bee families but highlights a loss in a Megachilidae species

**FIGURE 2:**
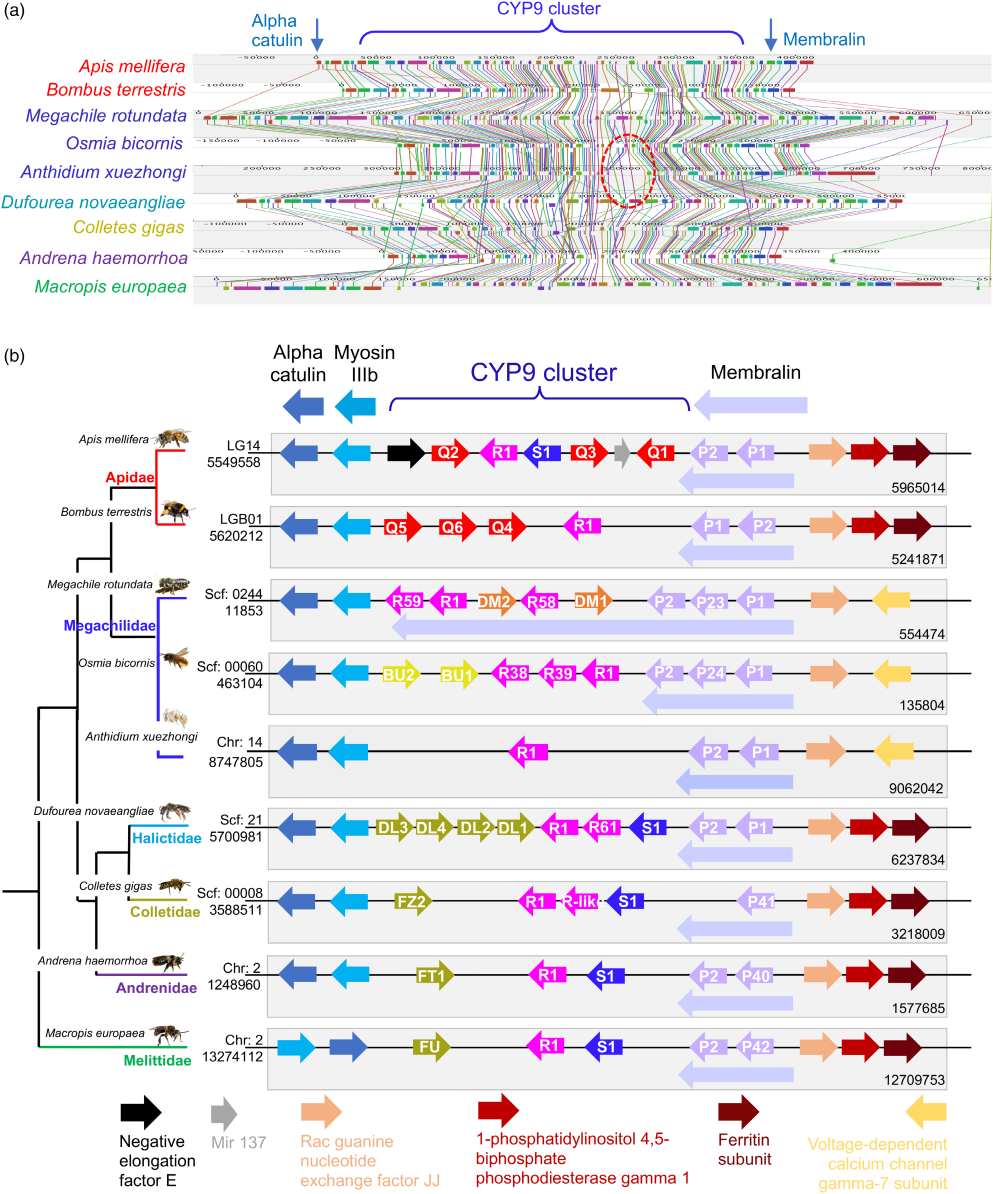
Syntenic relationship of the CYP9 loci in nine bee species across the six main bee families. (a) Locally collinear blocks (LCBs) located at the CYP9 loci in nine species across the six bee families. Each coloured block is a region without rearrangement of homologous backbone sequence (a collinear block). Lines drawn between sequences trace orthologous LCBs through the genomes. The red dashed‐ellipse highlights a loss of syntenic genes in the Megachilidae species *Anthidium xuezhongi*. (b) Schematic representation of the syntenic relationship at the CYP9 loci in nine species across the six bee families. Arrows represent syntenic genes and denote reading frame (not drawn to scale). CYP9 genes are coloured by lineage: *CYP9P* purple, *CYP9R* pink, *CYP9S* blue, *CYP9DM* orange and the *CYP9Q*‐related genes: *CYP9Q* red, *CYP9BU* yellow, *CYP9DL/FZ/FT/FU* gold.

Syntenic analyses offer a useful augmentation of sequence‐based phylogenetics. Recent molecular studies now place the Melittidae as a sister group to the other bee families and so the inclusion of the *M. europea* genome (Falk et al., 2022) in our analyses allows us to resolve the ancestral genomic architecture of the CYP9 locus (Branstetter et al., 2017; Peters et al., 2017). Eight good‐quality bee genomes were used alongside that of *M. europea*, comprising 2 Apidae, 3 Megachilidae, 1 Halictidae, 1 Andrenidae and 1 Colletidae species. Scaffolds containing the CYP9 locus from each assembly were examined for evidence of microsynteny (Figure 2).

The Melittidae diverged from the other bee families approximately 149 million years ago, and as such, it appears that the CYP9 locus is well conserved, with uniformity in both gene order and orientation across this evolutionary timescale (Figure 2b). There is clear evidence of one incidence of synteny breakage and genomic rearrangement in the Megachilidae. This synteny break results in *rac guanine nucleotide exchange factor JJ* becoming flanked by *voltage‐dependent calcium channel gamma‐7 subunit* (Figure 2b), with the genomic rearrangement of *ferritin subunit* and *1‐phosphatidylinositol 4,5‐biphosphate phosphodiesyterase gamma 1* to a different scaffold. This rearrangement must have occurred after the divergence of the Megachilidae. However, in all species *membralin* is found in association with *CYP9P* genes. There has been an inversion event in the flanking genes *myosin IIIb* and *alpha‐catulin* between the Melittidae and the other bee families. However, the analyses only include a single Melittidae species and so it is unclear whether this event occurred before or after the divergence of the bee families.

In general, the *CYP9Q*‐related genes show a high degree of conservation, both in genomic position and gene orientation. Indeed, only one *CYP9Q*‐related gene, *A. mellifera CYP9Q1*, appears to have undergone an inversion. The Melittidae, Colletidae and Andrenidae species all have a single *CYP9Q‐*related gene, whereas the Apidae and Halictidae have an expanded repertoire of three and four genes respectively. The biggest differences in the CYP9 subfamily appear to occur in the Megachilidae. Of the three species included, only *O. bicornis* has any *CYP9Q‐*related genes (two *CYP9BU* sequences). *M. rotundata* does have two *CYP9DM* genes that could be considered as syntenic substituents, but these sequences are distinct from a phylogenetic point‐of‐view as they cluster with the *CYP9R* and *CYP9S* lineages. Moreover, the CYPome of the third Megachilidae species, *A. xuezhongi*, contains only three *CYP9* genes in total: two *CYP9P*s and one *CYP9R*, and shows no evidence of either a *CYP9BU*‐ or *CYP9DM*‐related gene. These results further strengthen the implication that the selection acting on this important subfamily of P450s, within certain tribes of Megachilidae, appears to have created atypical and unique sets of detoxification enzymes.

### 3.4 Functional characterization of CYP9 sequences reveals divergence in the capacity of CYP9 enzymes from the Megachilidae to metabolize neonicotinoid insecticides

To examine the capacity of the Megachilidae *CYP9BU* and *CYP9DM* gene lineages, to metabolize insecticides that are efficiently detoxified by CYP9Q‐type P450s in other species, nine exemplar sequences were selected for functional expression comprising *M. rotundata* CYP9DM1 and CYP9DM2, *C. florisomne* CYP9BU3, *D. cincta* CYP9BU4, *H. truncorum* CYP9BU5 and *O. lignaria* CYP9BU1, *H. truncorum* CYP9BU6, *O. bicornis* CYP9BU1 and CYP9BU2 (Figure S2). All candidate genes possess the five conserved motifs common to insect P450s (Figure S2). Using a baculovirus and insect cell line system we successfully expressed recombinant proteins for all P450s except *H. truncorum* CYP9BU5, which was not successfully expressed as evidenced by a lack of a 450 nm peak in the CO‐difference spectra and an absence of activity to coumarin substrates, and was therefore excluded from further analyses (Table S4).

**FIGURE 3:**
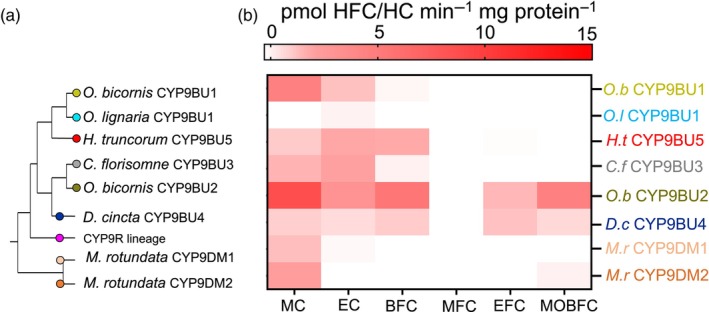
Heat map displaying the coumarin fluorophore substrate profile of eight recombinantly expressed CYP9BU P450s from six Megachilidae bee species. (a) Schematic of the phylogenetic relationship between the CYP9BU genes (b) Metabolism of selected coumarin results in fluorescent 7‐hydroxy coumarin (MC and EC) and 7‐hydroxy‐4‐(trifluoromethyl)‐coumarin (BFC, MFC, EFC, MOBFC) product respectively. Data are mean values (*n* = 3). BFC, 7‐benzyloxy‐4‐(trifluoromethyl)‐coumarin; EC, 7‐ethoxy‐coumarin; EFFC, 7‐ethoxy‐4‐(trifluoromethyl)‐coumarin; MC, 7‐methoxy‐coumarin; MFC, 7‐methoxy‐4‐(trifluoromethyl)‐coumarin; MOBFC, 7‐(4‐methoxybenzyloxy)‐4‐trifluoromethyl‐coumarin.

To gain an initial understanding of the substrate profile of the expressed P450s the recombinant CYP9 proteins were screened against a panel of fluorescent coumarin‐based model substrates. All eight of the proteins were found to be metabolically active against at least one coumarin‐based model substrate (Figure 3; Figure S3). In general, all the Megachilidae CYP9s exhibited a preference for smaller, non‐fluorinated coumarins with seven of the eight proteins showing significant activity towards MC and six towards EC. There was evidence for a secondary preference among the CYP9BU lineage towards activity against the larger benzylated coumarins such as BFC and MOBFC. In contrast, the CYP9DM lineage showed very little activity to any of the larger fluorinated coumarins. None of the recombinant CYP9s showed activity towards the O‐alkylated coumarin MFC.

**FIGURE 4:**
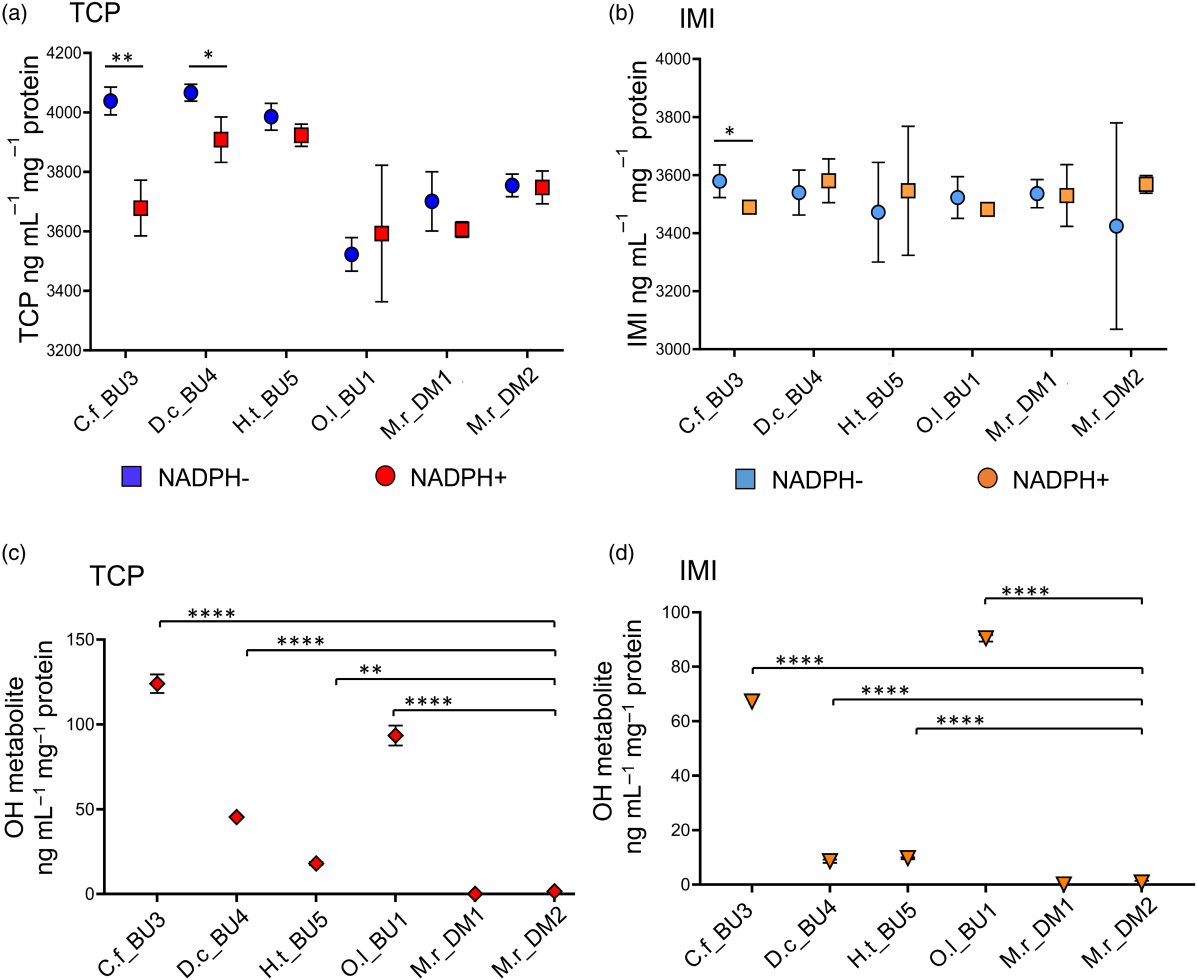
Metabolic profile of recombinantly expressed CYP9 P450s from bee species in the Megachilidae family screened against the neonicotinoid insecticides thiacloprid and imidacloprid. (a) TCP depletion by recombinantly expressed CYP9 P450s from the Megachilidae family of bees. TCP measured after 2 h by UPLC‐MS/MS and expressed as ng mL^−1^ mg^−1^ protein. (b) IMI depletion by recombinantly expressed CYP9 P450s from the Megachilidae family of bees. IMI measured after 2 h by UPLC‐MS/MS and expressed as ng mL^−1^ mg^−1^ protein. For (a) and (b) data are mean values ± S.D. (*n* = 3) and asterisks indicate significant depletion compared to controls without NADPH (*p* < 0.05, unpaired Welch's *t*‐test). (c) TCP OH‐metabolite production by recombinantly expressed CYP9 P450s from the Megachilidae family of bees. TCP‐OH measured after 2 h by UPLC‐MS/MS and expressed as ng mL^−1^ mg^−1^ protein. (d) IMI OH‐metabolite production by recombinantly expressed CYP9 P450s from the Megachilidae family of bees. IMI‐OH measured after 2 h by UPLC‐MS/MS and expressed as ng mL^−1^ mg^−1^ protein. For (c) and (d) data are mean values ± S.D. (*n* = 3) and asterisks indicate significant metabolite production compared across the two lineages of enzymes (CYP9BU and CYP9DM) using ANOVA and post‐hoc Dunnett's multiple comparison tests. Abbreviated CYP9 P450 names: *C. florisomne* CYP9BU3 (C.f_BU3), *D. cincta* CYP9BU4 (D.c_BU4), *H. truncorum* CYP9BU5 (H.t_BU5), *O. lignaria* CYP9BU1 (O.l_BU1), *M. rotundata* CYP9DM1 (M.r_DM1) and *M. rotundata* CYP9DM2 (M.r_DM2).

Ultra‐performance liquid chromatography–tandem mass spectrometry (UPLC–MS/MS) was then used to examine the capacity of the expressed bee P450s to metabolize two insecticides: imidacloprid (IMI) and thiacloprid (TCP) as exemplars of *N*‐nitroguanidine and *N*‐cyanoamidine neonicotinoids respectively. The two *O. bicornis* CYP9BU enzymes were excluded from these analyses because their capacity to metabolize neonicotinoid insecticides, specifically TCP and IMI, has been demonstrated previously (Beadle et al., 2019). Two of the recombinant P450s showed significant depletion of TCP compared to the controls (run without NADPH), with *C. florisomne* CYP9BU3 and *D. cincta* CYP9BU4 showing the greatest depletion values of 360 ng mL^−1^ mg^−1^ protein (*p* < 0.01) and 157.9 ng mL^−1^ mg^−1^ protein (*p* < 0.05) respectively (Figure 4a; Table S5). No significant depletion of TCP was detected for *O. lignaria* CYP9BU1, *H. truncorum* CYP9BU5 or either of the CYP9DM enzymes (Figure 4a; Table S5). There were significant differences in the level of TCP‐OH observed between all of the CYP9BU and both CYP9DM enzymes (ANOVA *p* < 0.0001; *post‐hoc* Dunnett's multiple comparison test *p* value range 0.0003 to <0.0001) (Figure 4c; Table S6). *C. florisomne* CYP9BU3, *H. truncorum* CYP9BU5 and *D. cincta* CYP9BU4 produced 123.97, 93.4 and 45.33 ng mL^−1^ mg^−1^ protein, respectively. This contrasts with the extremely low levels observed for CYP9DM1 and CYP9DM2 (<LOQ and 1.56 ng mL^−1^ mg^−1^ protein). Although, *O. lignaria* CYP9BU1 did not produce a significant decrease in TCP content, it did show significantly higher TCP‐OH production compared to the two CYP9DM enzymes (*p* < 0.0001), having the second highest value at 93.4 ng mL^−1^ mg^−1^ protein. *H. truncorum* CYP9BU5 showed the lowest level of TCP depletion compared to the control (62.28 ng mL^−1^ mg^−1^ protein) and the lowest metabolite production of the CYP9BU enzymes (17.97 ng mL^−1^ mg^−1^ protein). However, in the case of assays with *M. rotundata* CYP9DM enzymes, no production of the TCP‐OH metabolite was detected for CYP9DM1 and minimal levels for CYP9DM2 (1.56 ng mL^−1^ mg^−1^ protein) indicating that these P450s lack the capacity to substantially detoxify this insecticide. Furthermore, all of the CYP9BU enzymes showed significant metabolism of TCP when compared to *M. rotundata* CYP9DM1 and CYP9DM2 when considering production of known hydroxy‐metabolites (Table S6). In the case of IMI, only *C. florisomne* CYP9BU3 exhibited significant depletion of the compound in the presence of NADPH compared to the no‐NADPH control (*p* < 0.05), showing a depletion of 345.55 ng mL^−1^ mg^−1^ protein (Figure 4b; Table S5). The range of hydroxy‐imidacloprid (IMI‐OH) produced (0.72–90.43 ng mL^−1^ mg^−1^ protein) was generally lower than that seen with TCP‐OH (1.57–123.97 ng mL^−1^ mg^−1^ protein) (Figure 4c,d; Table S5). Indeed, the levels of IMI‐OH produced are significantly lower than those of TCP‐OH for three of the CYP9BU enzymes, *C. florisomne* CYP9BU3 (*p* < 0.01), *D. cincta* CYP9BU4 (*p* < 0.001) and *H. truncorum* CYP9BU5 (*p* < 0.01) (Table S4). Overall, these data show that some CYP9BU enzymes may be capable of metabolizing IMI, in addition to TCP, albeit at a lower efficiency. No significant production of IMI‐OH was observed for *M. rotundata* CYP9DM1 and CYP9DM2 (<LOQ and 0.72 ng mL^−1^ mg^−1^ protein, respectively) (Figure 4d).

In summary, these data reveal that, in general, the CYP9BU lineage of enzymes share a functional substrate profile with that of the CYP9Q‐related proteins found in species across the other bee families (Beadle et al., 2019; Haas et al., 2022), in that they show a preference for the *N*‐cyanoamidine neonicotinoid TCP over the *N*‐nitroguanidine neonicotinoid IMI. They also reveal that the *Megachile*‐specific CYP9DM enzymes do not have the capacity to detoxify these insecticides.

### 3.5 Modelling of bee CYP9 P450 proteins sheds light on different structural determinants for access tunnels involved in ligand transportation and binding

**FIGURE 5:**
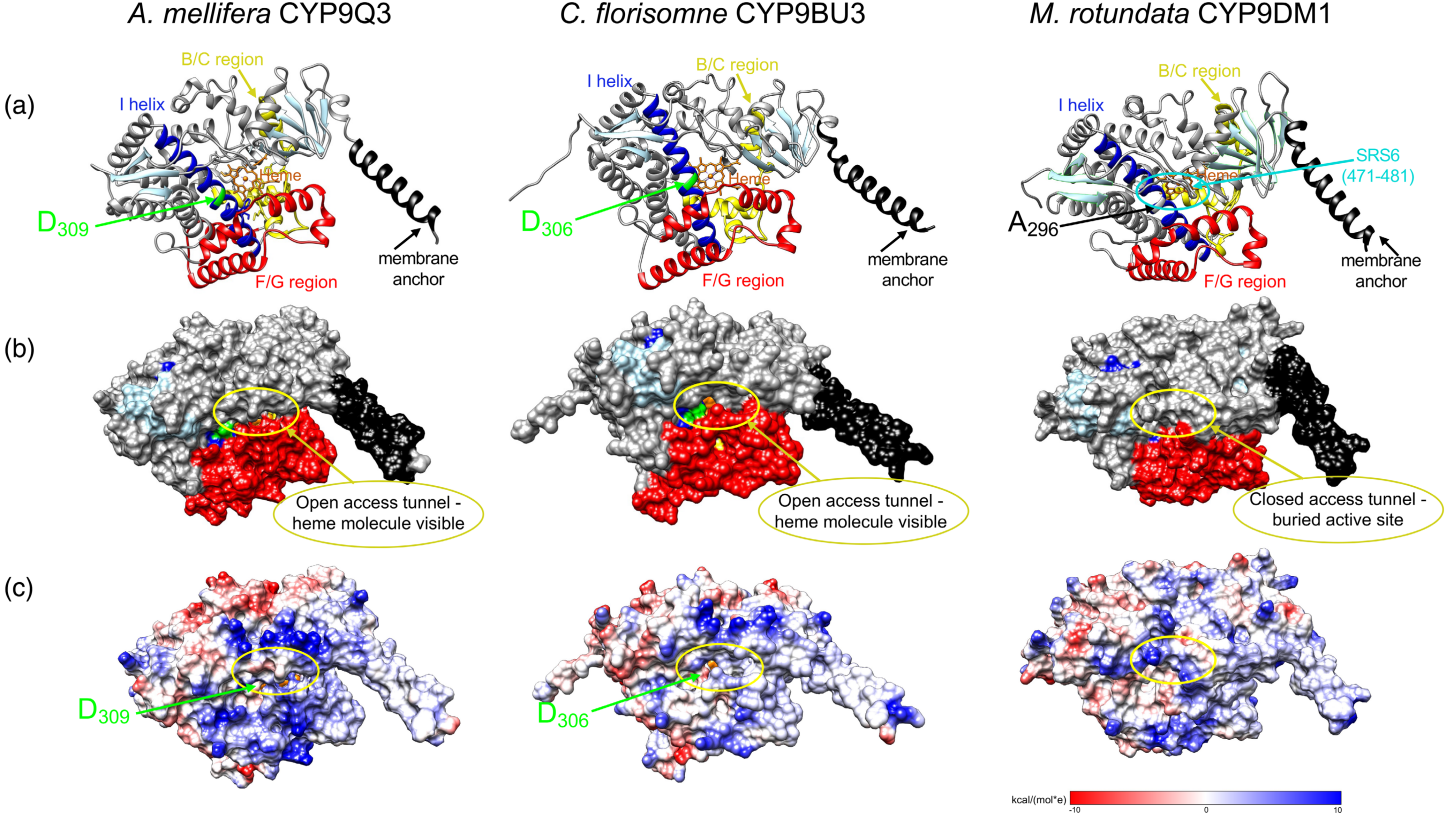
Tertiary structures related to substrate access tunnels and surface electrostatic potential of *A. mellifera* CYP9Q3, *C. florisomne* CYP9BU3 and *M. rotundata* CYP9DM1 protein sequences. (a) Secondary structure of proteins shown represented as cartoons. a‐helices coloured dark grey and b‐sheets coloured light blue apart from the B/C region, F/G region, I helix, membrane anchor and heme molecule which are coloured yellow, red, dark blue, black and orange, respectively. SRS4/M2 motif residues D309 (CYP9Q3) and D306 (CYP9BU3) shown in green and A296 (CYP9DM1) shown in white. The folding of SRS6 across the entrance to the access tunnel in CYP9DM1 is marked with a cyan ellipse. (b) Proteins shown with surface displayed, structure coloured as before. (c) Protein surface overlaid with electrostatic potential. Putative position of access tunnels entrance in (b) and (c) marked with yellow ellipse. Figure created using UCSF Chimera version 1.16.

To investigate the structure–function determinants of neonicotinoid metabolism three‐dimensional computational models of *A. mellifera* CYP9Q3 (known to bind/metabolize TCP), *M. rotundata* CYP9DM1 (known not to bind/metabolize TCP) and *C. florisomne* CYP9BU3 (shown to bind/metabolize both TCP and IMI) proteins were generated using AlphaFold2 (Jumper et al., 2021; Mirdita et al., 2022). The models obtained were of high quality as indicated by high ERRAT scores (93.5%, 97.4% and 89.1% for CYP9Q3, CYP9DM1 and CYP9BU3, respectively) and Ramachandran plots (Figure 5; Figure S4).

All three proteins achieved a druggability score of >0.8 rating them as druggable (Table S7). A large range in size of catalytic pocket between the three proteins was observed (1407–3043 Å^3^), with the known insecticide‐metabolizing CYP9Q3 having the smallest pocket volume (Table S7). TCP and IMI were used as ligands in a transport analysis and the most relevant tunnel for each was selected based on throughput, activation energy (*E*_a_) and highest binding energy (*E*_max_) values (Table S8). Tunnels 2 and 1 were selected in the case of CYP9Q3 and CYP9BU3 respectively (Table S9). However, a high energy barrier to binding either compound was observed for all four access tunnels for CYP9DM1, with positive *E*_max_ (range: 8.5–30.2 kcal/mol) and *E*_a_ values (range: 11.2–24.3 kcal/mol). So, despite negative *E*_bound_ values (−5.5 to −6.4 kcal/mol) it is unlikely there would be adequate transport through any of the tunnels that would allow the ligand to reach the active site of the protein (Table S10). However, for the sake of comparison, the tunnel with the lowest *E*_max_ and *E*_a_ values is included in Table S8 (tunnel 1).

The B/C and F/G regions of the predicted structures of *A. mellifera* CYP9Q3, *C. florisomne* CYP9BU3 and *M. rotundata* CYP9DM1 are shown in Figure 5a and Figure S5. CYP9DM1 has fewer aromatic amino acid residues present in these key regions (*n* = 13) than CYP9Q3 (*n* = 22) or CYP9BU3 (*n* = 21). The reduction in aromatic residue content may result in the F/G region of CYP9DM1 being less flexible and therefore less able to create conformation change to allow multiple substrates to bind. The protein surface topology of CYP9DM1 also differs from that seen in either CYP9Q3 or CYP9BU3, with the region containing SRS6 (residues 471–481) folding across the molecule to come into contact with helix‐A and the F/G region to form a less open conformation and bury the active site (Figure 5a,b).

These changes in protein topology and amino acid content have created a different surface electrostatic potential in CYP9DM1 when compared to CYP9Q3 or CYP9BU3 (Figure 5c). In particular, the substitution of D296A in conserved motif two of CYP9DM1 changes the electrostatic charge at the entrance to the access tunnel, effectively removing the negatively charged (red) lining of the inner part of the tunnel (Figure 5c). The exposed surface residues at the entrance to the access tunnel are key to the initial docking of substrates (Ravichandran et al., 1993) and so the difference in electrostatic charge may indicate that CYP9DM1 targets a distinctive group of substrates from the other two CYP9s.

Together these results reveal candidate structure–function determinants of neonicotinoid metabolism that may explain the metabolic differences observed between these distinct lineages of CYP9 P450s. Two major factors in whether these enzymes have the potential to bind multiple substrates, and thereby play a prominent role in response to environmental toxins, might be whether the active site is buried or open, and/or whether composition of the F/G region and access tunnels allow for conformational flexibility of the protein. The presence of a negatively charged residue in conserved motif two (e.g. D_309_: CYP9Q3, D_306_: CYP9BU3) may also be involved in the electrostatic steering of a substrate towards the opening of the access tunnel.

## 4 DISCUSSION

Our data provide new insights into the evolution of the CYP9 P450 family in the Megachilidae and the extent to which insecticide‐detoxifying P450s related to the CYP9Q subfamily are present in this important family of bees.

Our analyses confirm that the presence of *CYP9Q*‐related P450s is highly conserved across the diversity of bees, with the CYPomes of all species belonging to five out of the six families of bee included in the phylogenetic analyses, containing at least one *CYP9Q* ortholog. Importantly, however, it is clear from our phylogenetic analyses that several Megachilidae species lack *CYP9Q*‐related genes. *Coelioxys conoidea*, *Lithurgus chrysurus*, *Stelis punctulatissima*, the two *Anthidium* species and all five *Megachile* species do not have *CYP9Q‐*related sequences. Although most of these species belong to the subfamily Megachilinae, (*L. chrys*urus alone belongs to the Lithurginae), they can be differentiated from each other at the level of tribe (Gonzalez et al., 2012; Michener, 2007). Those species lacking *CYP9Q‐*related genes fall into three tribes: the Lithurgini, Megachilini and Anthidini. The Megachilidae species that have *CYP9BU* genes are from the Osmiini and Dioxyini tribes.

In contrast, each of the four Melittidae species included in our phylogeny has a single *CYP9Q*‐related gene and, given that this family is a sister group to all other bees, we can be confident that the presence of this lineage pre‐dates the divergence of bee families (Branstetter et al., 2017; Hedtke et al., 2013). Two of these Melittidae P450s have been functionally expressed (*Macropis fulvipes CYP9FU2* and *Melitta haemorrhoidalis CYP9FU3*) and both show the conserved capacity to metabolize certain synthetic insecticides common to the *CYP9Q*‐related lineage (Haas et al., 2022). The inclusion of *M. europaea* in the syntenic analyses in our study indicates that, in keeping with other bee species, its *CYP9Q*‐related sequence is found in a CYP9 gene cluster, in association with *alpha‐catulin* and *myosin IIIb*. Overall, these data allow us to predict that the ancestral, proto‐bee would have had at least one *CYP9Q*‐related gene, located in a CYP9 gene cluster similar to that found across most bee families today. It also seems probable that this ancestral CYP9 P450 had the functional capacity to metabolize a diverse number of naturally occurring xenobiotics and was pre‐adapted to detoxify certain synthetic insecticides.

We find that, in common with *CYP9Q*‐related P450s from other bee families and *O. bicornis CYP9BU1*, where *CYP9BU*‐type P450s are present in Megachildae species, they show the capacity to metabolize thiacloprid (Beadle et al., 2019; Haas et al., 2022). It is not unreasonable to predict that, in keeping with other *CYP9Q* and *CYP9BU* P450s, they are also likely to be pre‐adapted to metabolize other synthetic insecticides such as the butenolide flupyradifurone (Haas et al., 2022). Our results also reveal that the CYP9BU P450s expressed in this study are capable of binding multiple model substrates which implies that, in keeping with other *CYP9Q*‐related genes, they are likely to have broad substrate specificity, making them strong candidates for classification as xenobiotic‐metabolizing P450s (Denisov et al., 2009; Korzekwa et al., 1998).

All five *Megachile* species have evolved phylogenetically distinct, divergent CYP9 sequences in the place of *CYP9Q*‐reated orthologs, the *CYP9DM* lineage. While the *CYP9DM* genes are phylogenetically divergent, they show a good syntenic relationship to their *CYP9Q*‐related counterparts from other bee species. Our syntenic analyses also confirm the complete lack of either *CYP9Q‐* or *CYP9DM‐*related genes in *A. xueuzhongi* (Anthidiini), which only has three CYP9 genes in total. *M. rotundata* has been shown to have a high in vivo sensitivity to thiacloprid and an inability to metabolize this compound in vitro (Hayward et al., 2019). The species has also been shown to have a high in vivo sensitivity to the other insecticides known to be metabolized by CYP9Q and CYP9BU P450s (Table S11) (Beadle, 2018; Hayward, 2021). This knowledge, combined with the data from phylogenetic and syntenic analyses, strongly suggests that *CYP9DM* genes have lost the capacity to detoxify these compounds, as a direct consequence of divergent evolution. The current study provides clear evidence that this is indeed the case, with functional expression of CYP9DM1 and CYP9DM2 of *M. rotundata* demonstrating that both P450s lack the capacity to detoxify both imidacloprid and thiacloprid. It is likely that the four other *Megachile* species in this study, all of which have *CYP9DM* genes rather than *CYP9Q* orthologs, share the inability to metabolize these compounds and consequential high sensitivity to them. The complete absence of either *CYP9Q‐* or *CYP9DM‐*related genes in *A. xueuzhongi* and *A. manicatum* suggest these species, and perhaps genus, also lack the pre‐adaptation to tolerate certain synthetic insecticides.

Births and deaths within subfamilies of P450s are not uncommon events. Indeed, gene duplication followed by neo‐functionalization, or sub‐functionalization, has been linked to the augmentation of the metabolic profile of a species and also to host‐switching in herbivorous insects (Darragh et al., 2021; Feyereisen, 2011; Shi et al., 2021; Wang et al., 2018). An example of this is the loss of the *CYP6B* P450 subfamily in certain Lepidopteran taxa. This subfamily is involved in detoxification of xenobiotics but almost a third (10/34) of the Lepidopteran species studied were documented to be lacking *CYP6B* genes (Shi et al., 2021). Whereas, the *CYP6B* subfamily has undergone a remarkable bloom of up to 26 members in certain other species (Shi et al., 2021). In Papilionidae butterflies the *CYP6B* subfamily has the conserved ability to metabolize furanocoumarins (Mao et al., 2007). However, the range of furanocoumarins that can be detoxified, and the rate of their metabolism by different *CYP6B* genes varies, and both measures are correlated to the breadth of host plant used by the species (Mao et al., 2007). In bees, P450s in the CYP3 clan, which contains many genes involved in the metabolism of xenobiotic compounds, have been shown to be more likely to undergo duplication or deletion events and appear to be under dynamic selection pressures (Darragh et al., 2021). Indeed, members of the *CYP9R* and *CYP9Q* lineages are considered to be unstable and to exhibit signs of adaptive evolution (Darragh et al., 2021).

In addition to the *Megachile* and *Anthidium* genera, three other Megachilidae species lack a *CYP9Q* ortholog. Of these two, *C. conoidea* and *S. punctulatissima*, are cleptoparasitic ‘cuckoo’ bees, and as such do not collect pollen to provision nest cells for their offspring and therefore have reduced exposure to naturally occurring xenobiotics. However, in cuckoo bees that do not have a *CYP9Q* ortholog, the choice of a suitable host species is potentially a determining factor in larval survival. Nest provisions that require *CYP9Q*‐specific detoxification of secondary metabolites could theoretically be toxic to larvae without genes from this lineage. In general, cleptoparasitic bees tend to target close relatives, often species from the same genus or tribe (Bogusch et al., 2006; Michener, 2007) and this is indeed the case for *C. conoidea* and *S. punctulatissima*, which target host species from genera lacking a *CYP9Q*‐ortholog (Kasparek, 2015; Litman et al., 2013; Scott et al., 2000). Conversely, cleptoparasite species with a *CYP9Q‐*ortholog, such as *N. lathburiana* (Apidae) and *D. cincta* (Megachilidae), target genera that also have similar orthologous genes, such as *Andrena* or *Osmia* (Haas et al., 2022; Rozen & Ozbek, 2005; Schindler et al., 2018). Thus, it appears that the dietary range of the parasite and the host complement each other.

The third species that lacks a *CYP9Q*‐ortholog, *L. chrysurus*, is thought to be a strict oligolectic species, relying almost exclusively on *Centaurea* (Asteraceae) pollen. A decrease in the breadth of host plants may reduce the number of plant protective allelochemicals encountered while foraging and subsequently lower the detoxification challenge to the species. However, it is unlikely that dietary breadth alone is the sole determining factor of the presence or absence of a *CYP9Q*‐related gene. Nonetheless, these data indicate that divergent selection acting upon certain Megachilidae genera has led to important differences in the repertoire of this key group of *CYP9* P450 detoxification genes.

*Megachile* species line their brood cells with discs of leaves or petals that they cut using their mandibles, and as such are commonly called true leafcutter bees. Whereas, *Anthidium* species scrape or cut trichomes (plant ‘hairs’), with their mandibles, to create their cotton‐like brood cells, a behaviour that gives rise to their common name of carder bees (Gonzalez & Griswold, 2013; Muller, 1996). By default, collecting nesting material by cutting leaves, or removing trichomes, actively damages the plant from which it is taken (Graham et al., 2017; Sinu & Bronstein, 2018). Plants have evolved complex biochemical defences to minimize damage from herbivorous insects, for example, *Arabidopsis thaliana* produces glucosinolates and anthocyanins in response to leaf vibrations caused by insect chewing (Appel & Cocroft, 2014). Whilst leafcutter and carder bees are not ingesting the plant material they collect, these behaviours cause tissue damage and will induce defence responses in the plant (Bonaventure, 2012; War et al., 2018). It is possible that exposure to a unique array of plant defence chemicals, and/or compounds at higher concentrations than encountered in pollen and nectar, might explain the evolution of the divergent, more specialized repertoire of detoxification enzymes observed in *Megachile* and *Anthidium* species.

Our analyses of the 3D structures of three exemplar P450s, *A. mellifera* CYP9Q3, *C. florisomne* CYP9BU3 and *M. rotundata* CYP9DM1 in this study revealed potential determinants associated with the divergent insecticide metabolism by CYP9DM versus CYP9Q‐related P450s. The first key difference between these proteins appears in the oxygen‐binding motif, M2 (conserved motif: G‐x‐E/D‐**T**‐T/S), where the charged acid residue (aspartic/glutamic acid; E/D) is lost in the CYP9DM protein. The substitution of the anionic amino acid (E/D) for a non‐charged residue, either alanine (A: hydrophobic) or threonine (T: polar) at the centre of the oxygen‐binding motif of the CYP9DM enzymes would be predicted to significantly alter the binding of that portion of the active site. This substitution also modifies the electrostatic charge at the protein surface, thereby potentially attracting a different set of substrates to the protein surface of CYP9DM1. Work on human CYP3A4, has demonstrated the importance of key amino acid residues in the SRSs, including E_308_ from the SRS4/M2 motif (Kiani et al., 2019). Further, a single charged residue in SRS4 (D_298_), in the CYP336A P450 family across the Hymenoptera, has also been shown to act as a gatekeeper that facilitates the specificity of the enzyme (Haas et al., 2023). As such, the loss of the anionic residue in the CYP9DM enzymes (equivalent to E_308_ in CYP3A4), provides an indication that these enzymes bind a different group of substrates to those of the CYP9Q‐related lineage. This is supported by our ligand transport analyses of TCP and IMI using CAVER and the AlphaFold model of CYP9DM1 which reveal the high energy barrier (positive *E*_max_ and *E*_a_ values) involved in transporting these neonicotinoids from the surface of CYP9DM1 to the active site.

Research on CYP3A4 has also revealed that the B/C and F/G regions of the protein are involved in substrate channel structure (Denisov et al., 2015; Fishelovitch et al., 2009; Skopalík et al., 2008). Aromatic amino acid residues in the F/G region of CYP3A4 are directly involved in controlling the opening and closing of substrate access channels (Denisov et al., 2015; Fishelovitch et al., 2009; Skopalík et al., 2008). In particular, rotation of the side chains of key phenylalanine residues in the F/G region of CYP3A4 allows this part of the protein to be highly flexible, changing the conformation of access from the protein surface to the active site (Skopalík et al., 2008). The B/C region, which contains the WxxxR conserved motif, is also key to the substrate channel structure of CYP3A4 (Fishelovitch et al., 2009). Our work demonstrates that, in comparison to CYP9Q3 and CYP9BU3, CYP9DM1 has fewer aromatic amino acid residues in both of these key regions. These differences may result in CYP9DM1 being less conformationally flexible and therefore less able to transport or bind multiple substrates.

Whilst the differences between the three CYP9 protein sequences do not prevent the enzymes from folding in a biologically relevant manner, there are marked differences in the overall topology of the predicted structures. Most notable is that the active sites in CYP9Q3 and CYP9BU3 appear to be open in form. Indeed, the heme molecule is visible at the bottom of the opening of the access tunnels (Figure 5b). In contrast, the active site of CYP9DM1 is buried, or exhibits a closed in form, in which the heme molecule cannot be seen from the protein surface. Active site structures in P450s have been classified as closed, holey or open, based on solvent accessibility and radius of access tunnel, with closed structures often blocked at the surface by an inward turn of the F/G region (Wade et al., 2004). Here, the access to the active site in CYP9DM1 is blocked at the surface by the region containing SRS6 (residues 471–481) folding across the molecule to come into contact with helix‐A and the F/G region (Figure 5a). This is relevant as the extent to which the access channel of a P450 can open is thought to correlate with whether they have the capacity to bind multiple, diverse substrates, or are more targeted, specific enzymes (Urban et al., 2018; Wade et al., 2004).

These data raise concerns for the safe use of insecticides, not only for the pollination services provided by managed species, such as *M. rotundata*, but also for the health of wild species from the Megachilidae in general. While the value of the pollination service attributed to this bee family is unknown, calculations of the pollination service value of wild‐bees to the US economy put the figure at over $3 billion per annum (Calderone, 2012; Danforth et al., 2019). Given this, it is important to conduct further in vivo work and acute toxicity testing to confirm whether, Megachilidae species lacking *CYP9Q*‐related orthologs, other than *M. rotundata*, are indeed more vulnerable to insecticides than other bees. However, standardized protocols for toxicity tests in solitary bee species are yet to be developed, and there are inherent difficulties in the collection of specimens for use in this sort of assessment (Lehmann & Camp, 2021). Given these implications, there is a need to develop standardized comparative genomics analyses and targeted in vitro functional tests, such as those described in this study, that can inform the current ecotoxicological trials, or be included as tier 0 tests. Acute contact toxicity testing could then be targeted at species or genera as required to help avoid negative outcomes of insecticide use.

In this regard, it is clear that the current use of *A. mellifera* as a proxy in ecotoxicological testing is unreliable for many Megachilidae species. The suggested use of an assessment or bridging factor of 10, to allow toxicological data from *A. mellifera* to be used in other species, as recommended by the European Chemical Agency in 2020, would also not reduce the risks to species such as *M. rotundata* to a safe level for certain insecticides (Ansell et al., 2021; Arena & Sgolastra, 2014; ECHA, 2020; Hayward et al., 2019; Pamminger, 2021). The suggestion that either *B. terrestris* or *O. bicornis* (or both) should be added as extra model species to the toxicity trials would also not give at risk Megachilidae taxa the protection they need (EFSA, 2013; Sgolastra et al., 2019, 2020; Uhl et al., 2019). A better choice might be that *M. rotundata* is added as the model solitary bee species, perhaps as part of a wider, case‐specific selection of model species. Not only does the species fulfil the criteria required from a new surrogate species for tier one testing of insecticides, but due to its use as a commercial pollinator, it is also readily available for testing.

## CONFLICT OF INTEREST STATEMENT

This study received funding from Bayer AG, a manufacturer of neonicotinoid and butenolide insecticides. J.H. and R.N. are employees of Bayer AG.

## Supporting information


Data S1:

Dataset S1:

## Data Availability

Raw sequence data and assemblies have been submitted to NCBI Short Read Archive and Transcriptome Shotgun Assembly databases under BioProject PRJNA962714. All other relevant data are provided in the supplementary information.
