# Supplementary material for: A cytochrome P450 insecticide detoxification mechanism is not conserved across the Megachilidae family of bees
Source: Evol Appl. 2023 Dec 6;17(1):e13625. doi: 10.1111/eva.13625 (PMC10810168; doi:10.1111/eva.13625)
Supplement: Supplementary file 1 — Data S1: [file EVA-17-e13625-s002.pdf]

A cytochrome P450 insecticide detoxification mechanism is not conserved across the Megachilidae family of bees

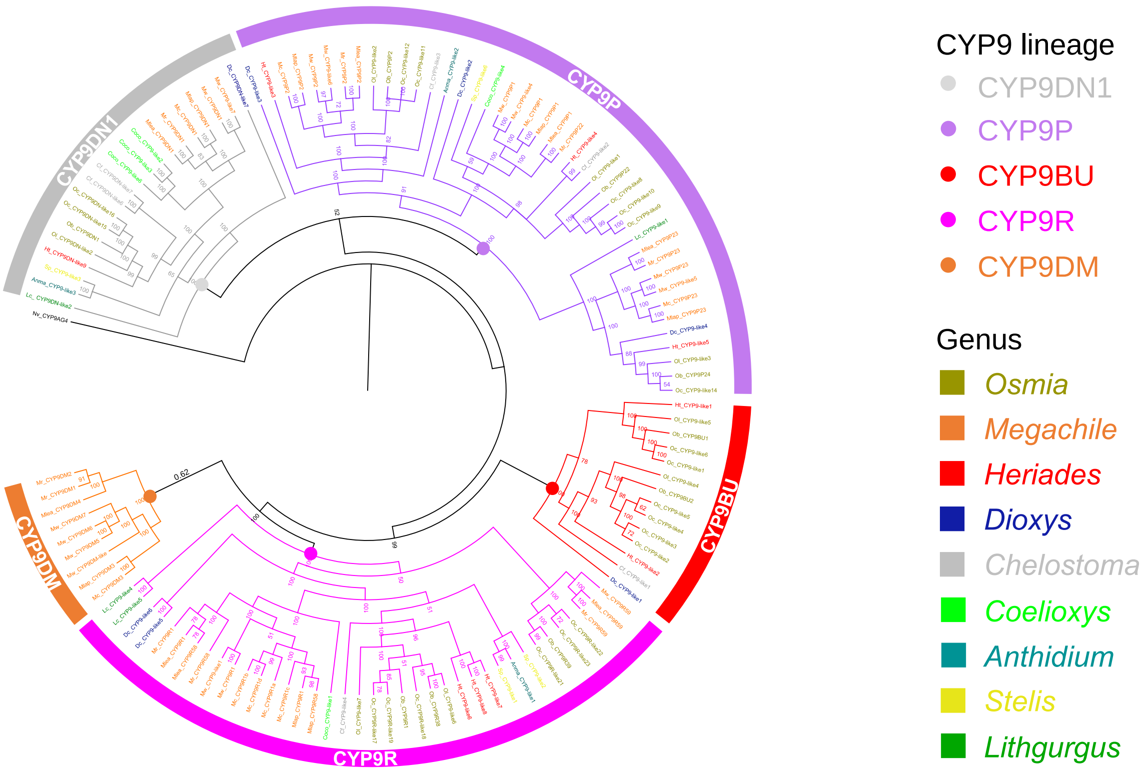

Figure S1: Phylogeny of the Megachilidae CYP9 P450s. Phylogenetic tree estimated using Bayesian inference. Node support is shown as % posterior probability. Tree is rooted on *Nasonia vitripennis* CYP9AG4 (NCBI reference sequence: NP_001166010.1). CYP9 sequences cluster to form distinct lineages: CYP9DN1 (grey), CYP9P (purple), CYP9R (pink), CYP9BU (red) and the *Megachile*-specific CYP9DM (orange). The ancestral node of each lineage is marked with a circle coloured by lineage. Outer leaves of CYP9 lineages are coloured by genus and annotated with an abbreviated form of species name. [Abbreviated species names: Mr – *Megachile rotundata*, Mc – *M. centuncularis,* Mlap – *M. lapponica, Mlea – M. leachella,* Mw – *M. willughbiella,* Ob – *Osmia bicornis,* Oc – *O. cornuta,* Ol – *O. lignarua,* Dc – *Dioxys cincta,* Ht – *Heriades truncorum,* Cf – *Chelostoma florisomne,* Anma – *Anthidium manicatum,* Sp – *Stelis punctulatissima,* Coco – *Coelioxys conoidea,* Lc – *Lithurgus chrysurus*]

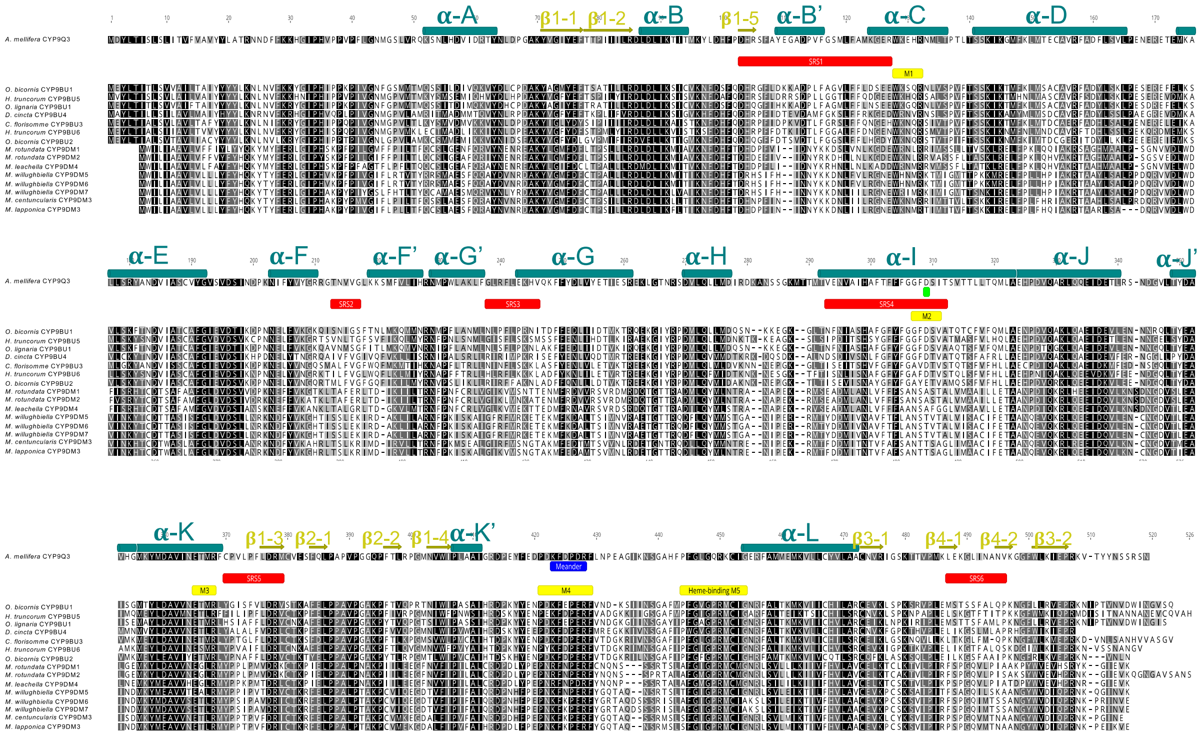

Figure S2: MSA of CYP9BU proteins from five Megachilidae species, CYP9DM proteins from five Megachile species and A. mellifera CYP9Q3 showing secondary structure, conserved motifs and SRSs

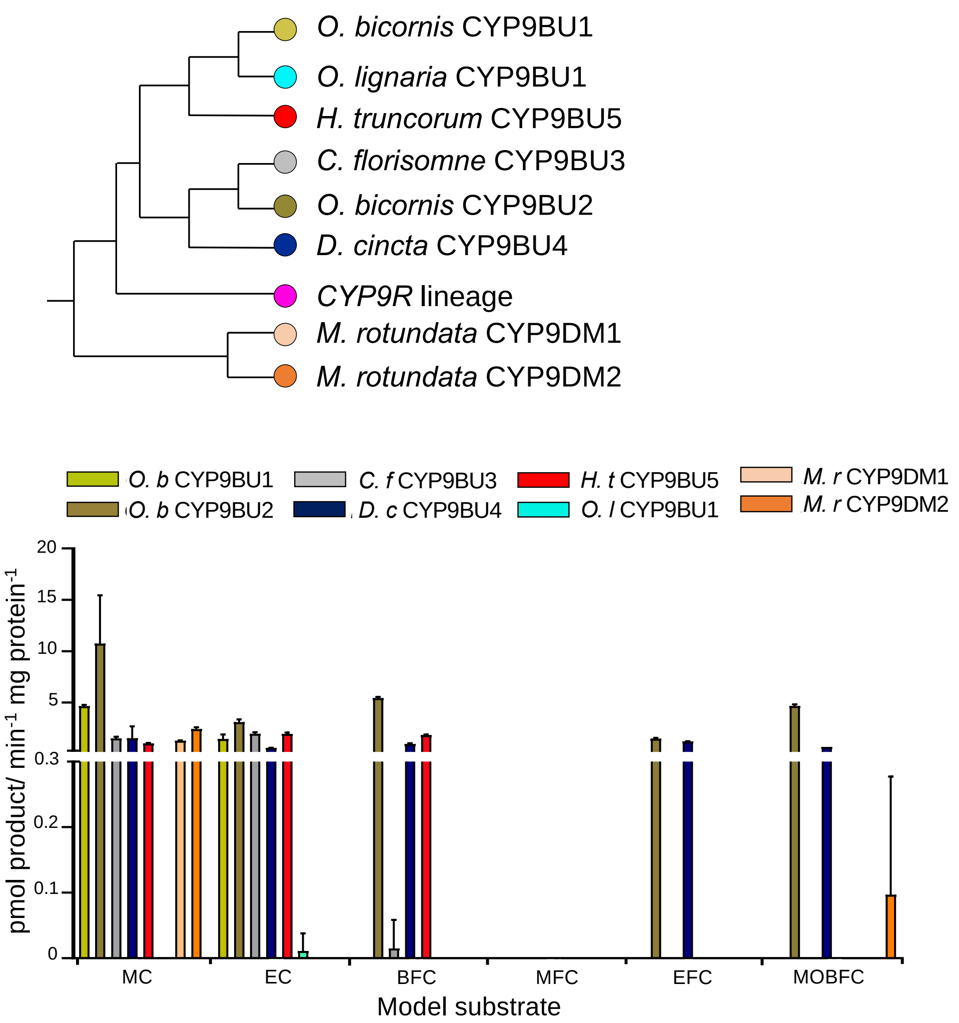

Figure S3: The coumarin fluorophore substrate profile of eight recombinantly expressed CYP9BU P450s from six Megachilidae bee species. (A) Schematic of the phylogenetic relationship between the CYP9BU genes. (B) Metabolism of selected coumarin results in fluorescent 7-hydroxy-4-(trifluoromethyl)-coumarin (BFC, MFC, EFC, MOBFC) and 7-hydroxy coumarin (MC and EC) product respectively. Data are mean values (n=3).

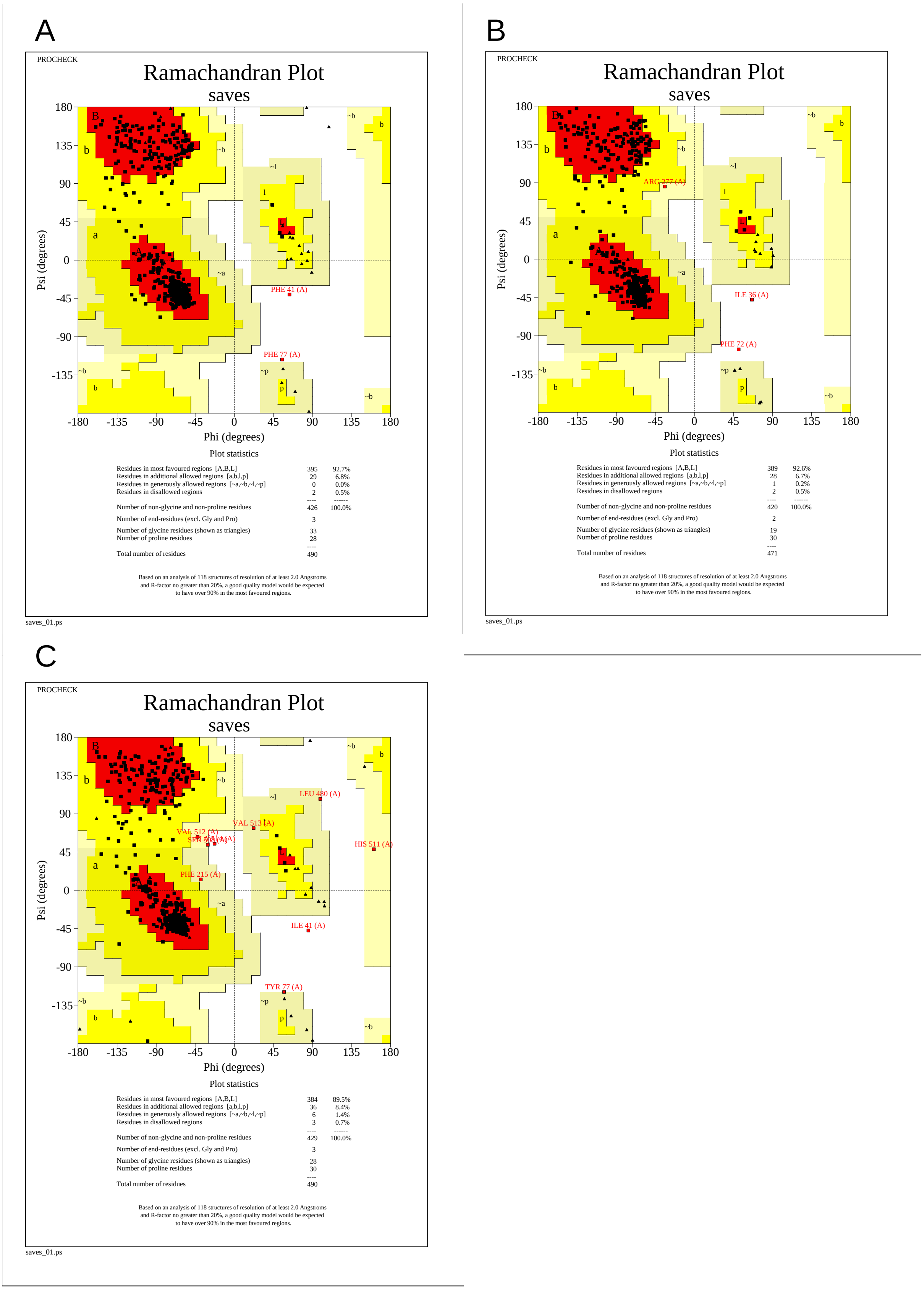

Figure S4: Ramachandran plots for three-dimensional computational models of CYP9 proteins generated using AlphaFold2.
(A) *A. mellifera* CYP9Q3. (B) *M. rotundata* CYP9DM1. (C) *C. florisomne* CYP9BU3.

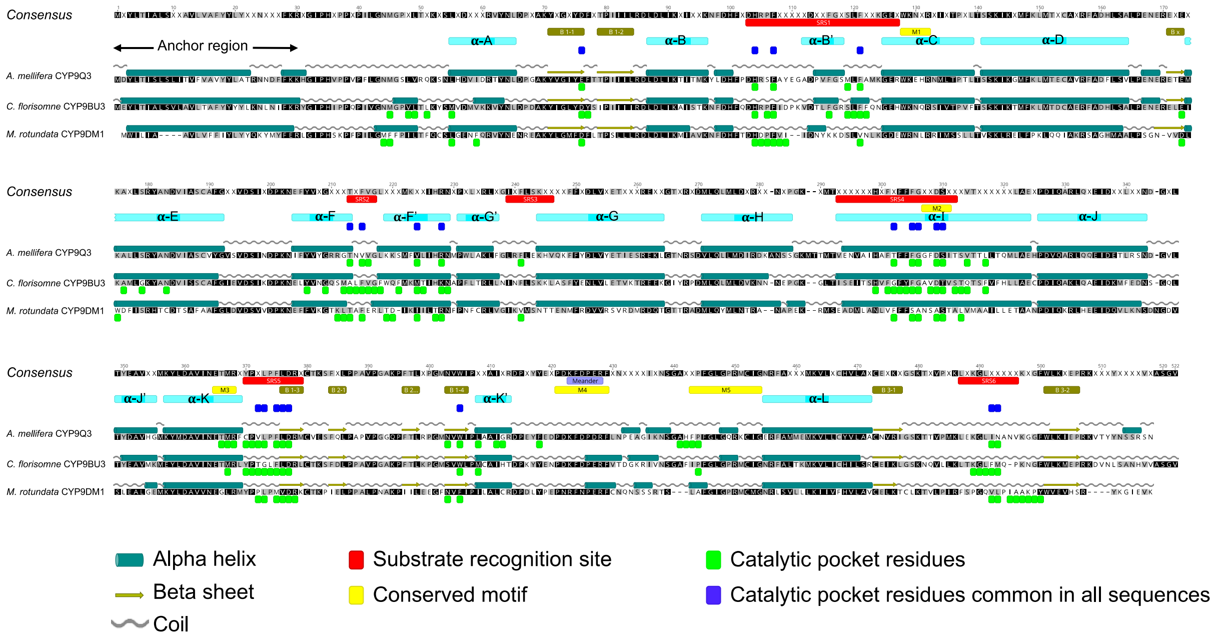

Figure S5: Multiple sequence alignment of *A. mellifera* CYP9Q3, *C. florisomne* CYP9BU3 and *M. rotundata* CYP9DM1 protein sequences. Aligned in Geneious version 10.2.6 (Biomatters) using MUSCLE (Edgar, 2004). The sequences are coloured black to white according to their similarity. Conserved motifs (M) and substrate recognition sites (SRS) are shown in the consensus annotated in yellow and red respectively. Secondary structure of the protein is shown in the consensus and above each protein, with alpha helices, beta sheets and coiled regions shown as dark cyan cylinders, gold cylinders/arrows and grey lines. Secondary structure marked with reference to the AlphaFold2 models predicted in this study. Residues that make up the catalytic pocket were predicted using CAVER Web v1.1 (Stourac *et al*., 2019) and are shown in green under each protein sequence. Catalytic pocket residues common to all sequences are annotated blue in the consensus.

Table S1: Assembly statistics for the transcriptomes of *M. centuncularis*, *M. leachella, M. lapponica* and *M. willughbiella*. The BUSCO results presented are based on analysis using the Insecta odb10 test gene set.

Metric	*Megachile centuncularis*	*Megachile leachella*	*Megachile lapponica*	*Megachile willughbiella*
No. sequences	185,210	241,588	148,465	204,567
No. of bases	355,169,076	430,502,408	372,071,194	372,899,138
Mean contig length (bp)	1,918	1,782	2,506	1,823
Contigs over 1kbp	68,856	88,047	68,044	73,415
Contigs with ORF	60,489	84,389	58,242	69,597
N50 (bp)	5375	4979	6342	5235
GC content	0.37833	0.38485	0.37644	0.37954
Peptides	29,620	44,616	24,386	37,365
% Complete BUSCOs (C)	99.5%	99.5%	99.2%	99.4%
% Complete and single-copy BUSCOs (S)	90.4%	90.1%	89.0%	89.7%
% Complete and duplicated BUSCOs (D)	9.1%	9.4%	10.2%	9.7%
% Fragmented BUSCOs (F)	0.1%	0.1%	0.3%	0.2%
% Missing BUSCOs (M)	0.4%	0.4%	0.5%	0.4%

Table S2: Comparison of the CYPomes of bee species across four families (Apidae, Megachilidae, Halictidae and Colletidae) with emphasis on the *Megachile* genus.

	Comparison of CYPomes from bee species									
Family	Megachilidae						Apidae		Halictidae	Colletidae
Species	*Megachile rotundata*	*Megachile centuncularis*	*Megachile lapponica*	*Megachile leachella*	*Megachile willughbiella*	*Osmia bicornis*	*Apis mellifera*	*Bombus terrestris*	*Dufourea novaeangliae*	*Colletes gigas*
**CYP3 Clan** CYP6s	CYP6AQ52					CYP6AQ55	CYP6AQ1	CYP6AQ26	CYP6AQ47	CYP6AQ77
	CYP6AQ53	CYP6AQ53a	CYP6AQ53	CYP6AQ53	CYP6AQ53a			CYP6AQ27	CYP6AQ48	CYP6AQ78
		CYP6AQ53b						CYP6AQ28	CYP6AQ49	
					CYP6AQ53c					
	CYP6AQ54	CYP6AQ54	CYP6AQ54	CYP6AQ54	CYP6AQ54			CYP6AQ29	CYP6AQ50	
								CYP6AQ31		
								CYP6AQ33		
	CYP6AS108	CYP6AS108	CYP6AS108	CYP6AS108	CYP6AS108	CYP6AS121	CYP6AS1	CYP6AS5	CYP6AS91	
	CYP6AS109	CYP6AS109	CYP6AS109	CYP6AS109	CYP6AS109	CYP6AS122	CYP6AS2	CYP6AS7	CYP6AS92	
	CYP6AS110	CYP6AS110	CYP6AS110	CYP6AS110	CYP6AS110	CYP6AS123	CYP6AS3	CYP6AS10	CYP6AS93	
	CYP6AS111	CYP6AS111	CYP6AS111	CYP6AS111	CYP6AS111	CYP6AS124	CYP6AS4	CYP6AS12	CYP6AS94	
	CYP6AS112	CYP6AS112	CYP6AS112	CYP6AS112	CYP6AS112	CYP6AS125	CYP6AS5	CYP6AS13	CYP6AS95	
	CYP6AS113	CYP6AS113	CYP6AS113a	CYP6AS113	CYP6AS113	CYP6AS126	CYP6AS5P	CYP6AS19	CYP6AS96	
			CYP6AS113b			CYP6AS127	CYP6AS7	CYP6AS72	CYP6AS97	
			CYP6AS113c			CYP6AS128	CYP6AS8	CYP6AS73	CYP6AS98	
	CYP6AS114					CYP6AS129	CYP6AS10	CYP6AS74	CYP6AS99	
	CYP6AS115	CYP6AS115	CYP6AS115	CYP6AS115	CYP6AS115	CYP6AS130	CYP6AS11	CYP6AS75	CYP6AS100	CYP6AS115
	CYP6AS116	CYP6AS116	CYP6AS116	CYP6AS116	CYP6AS116	CYP6AS131	CYP6AS12	CYP6AS76		
	CYP6AS117	CYP6AS117	CYP6AS117	CYP6AS117	CYP6AS117	CYP6AS132	CYP6AS13	CYP6AS77		
	CYP6AS118	CYP6AS118	CYP6AS118	CYP6AS118	CYP6AS118	CYP6AS133	CYP6AS14			
	CYP6AS119	CYP6AS119	CYP6AS119	CYP6AS119	CYP6AS119	CYP6AS134	CYP6AS15			
	CYP6AS120	CYP6AS120	CYP6AS120	CYP6AS120	CYP6AS120	CYP6AS135	CYP6AS16			CYP6AS120
						CYP6AS136	CYP6AS17			CYP6AS209
						CYP6AS151	CYP6AS18			CYP6AS210
							CYP6AS19			CYP6AS211
										CYP6AS212
	CYP6BC1	CYP6BC1	CYP6BC1	CYP6BC1	CYP6BC1	CYP6BC1	CYP6BC1	CYP6BC1	CYP6BC1	CYP6BC1
	CYP6BD1	CYP6BD1	CYP6BD1	CYP6BD1	CYP6BD1	CYP6BD1	CYP6BD1	CYP6BD1	CYP6BD1	CYP6BD1
	CYP6BE1	CYP6BE1	CYP6BE1	CYP6BE1		CYP6BE1	CYP6BE1	CYP6BE1	CYP6BE1	CYP6BE1
TOTAL	19	18	19	17	17	21	21	21	17	11
**CYP3 Clan** CYP336As	CYP336A33	CYP336A33	CYP336A33	CYP336A33	CYP336A33		CYP336A1	CYP336A22	CYP336A29	CYP336A56
								CYP336A23	CYP336A30	CYP336A57
								CYP336A24	CYP336A31	CYP336A58
	CYP336A34	CYP336A34	CYP336A34	CYP336A34	CYP336A34			CYP336A25	CYP336A32	CYP336A59
						CYP336A35				
						CYP336A36				
		CYP336A53								
		CYP336A54								
				CYP336A55						
	CYP336M1									
		CYP336L1	CYP336L1	CYP336L1	CYP336L1	CYP336L1				
TOTAL	3	5	3	4	3	3	1	4	4	4
**CYP3 Clan** CYP9s	CYP9DN1	CYP9DN1	CYP9DN1	CYP9DN1	CYP9DN1	CYP9DN1				
										CYP9DN2
										CYP9DN3
										CYP9DN4
										CYP9DN5
	CYP9P1	CYP9P1	CYP9P1	CYP9P1	CYP9P1	CYP9P1	CYP9P1	CYP9P1	CYP9P1	
	CYP9P2	CYP9P2	CYP9P2	CYP9P2	CYP9P2	CYP9P2	CYP9P2	CYP9P2	CYP9P2	
	CYP9P23	CYP9P23	CYP9P23	CYP9P23	CYP9P23	CYP9P24				CYP9P41
	CYP9DM1					CYP9BU1	CYP9Q1	CYP9Q4	CYP9DL1	
	CYP9DM2					CYP9BU2	CYP9Q2	CYP9Q5	CYP9DL2	
		CYP9DM3	CYP9DM3				CYP9Q3	CYP9Q6	CYP9DL3	
				CYP9DM4					CYP9DL4	
					CYP9DM5					CYP9FZ2
					CYP9DM6					
					CYP9DM7					
	CYP9R1	CYP9R1a	CYP9R1	CYP9R1	CYP9R1	CYP9R1	CYP9R1	CYP9R1	CYP9R1	CYP9R1
		CYP9R1b								
		CYP9R1c								
		CYP9R1d								
	CYP9R58		CYP9R58	CYP9R58		CYP9R38			CYP9R61	
	CYP9R59			CYP9R59	CYP9R59	CYP9R39				
							CYP9S1	CYP9S1	CYP9S1	CYP9S1
TOTAL	9	9	7	8	9	9	7	7	9	8
**CYP2 Clan**	CYP15A1	CYP15A1	CYP15A1	CYP15A1	CYP15A1	CYP15A1	CYP15A1	CYP15A1	CYP15A1	CYP15A1
	CYP18A1	CYP18A1		CYP18A1	CYP18A1 (across 3 contigs)	CYP18A1	CYP18A1	CYP18A1	CYP18A1	CYP18A1
	CYP303A1				CYP303A1 (across 2 contigs)	CYP303A1	CYP303A1	CYP303A1	CYP303A1	CYP303A1
	CYP305D1	CYP305D1	CYP305D1	CYP305D1	CYP305D1	CYP305D1	CYP305D1	CYP305D1	CYP305D1	CYP305D1
						CYP306A1	CYP306A1	CYP306A1	CYP306A1	CYP306A1
	CYP307B1	CYP307B1 (across 3 contigs)		CYP307B1	CYP307B1 (across 2 contigs)	CYP307B1	CYP307B1	CYP307B1	CYP307B1	CYP307B1
	CYP343A1					CYP343A1	CYP343A1			CYP343A1
	CYP369A1		CYP369A1	CYP369A1		CYP369A1	CYP369A1	CYP369A1	CYP369A1	CYP369A1
TOTAL	7	4	3	5	5	8	8	8	8	8
**CYP4 Clan**	CYP4G11	CYP4G11	CYP4G11	CYP4G11	CYP4G11	CYP4G11	CYP4G11	CYP4G11	CYP4G11	CYP4G11
	CYP4G202					CYP4G202			CYP4G202	CYP4AG323
	CYP4AA1	CYP4AA1	CYP4AA1	CYP4AA1	CYP4AA1	CYP4AA1	CYP4AA1	CYP4AA1	CYP4AA1	CYP4AA1
										CYP4AA15
						CYP4AB3	CYP4AB3	CYP4AB3	CYP4AB3	CYP4AB86
										CYP4AB87
										CYP4AB88
	CYP4AV1	CYP4AV1	CYP4AV1	CYP4AV1	CYP4AV1	CYP4AV1	CYP4AV1	CYP4AV1	CYP4AV1	
TOTAL	4	3	3	3	3	5	4	4	5	7
**Mitochondrial Clan**	CYP301A1	CYP301A1	CYP301A1	CYP301A1	CYP301A1	CYP301A1	CYP301A1	CYP301A1	CYP301A1	CYP301A1
	CYP301B1			CYP301B1 (across 3 contigs)		CYP301B1	CYP301B1	CYP301B1	CYP301B1	CYP301B1
	CYP302A1	CYP302A1		CYP302A1	CYP302A1	CYP302A1	CYP302A1	CYP302A1	CYP302A1	CYP302A1
	CYP314A1	CYP314A1	CYP314A1	CYP314A1	CYP314A1	CYP314A1	CYP314A1	CYP314A1		CYP314A1
	CYP315A1	CYP315A1		CYP315A1	CYP315A1	CYP315A1	CYP315A1	CYP315A1	CYP315A1	CYP315A1
	CYP334A1					CYP334A1	CYP334A1	CYP334A1	CYP334A1	CYP334A1
TOTAL	6	4	2	5	4	6	6	6	5	6

Table S3: Inventory of the lineages of the CYP9 subfamily of *A. mellifera, B. terrestris*, *D. novaeangliae, C. gigas, O. bicornis* and five species of *Megachile* bees.

Species (Family)	*CYP9DM*	*CYP9P*	*CYP9Q-related*	*CYP9R*	*CYP9S*	TOTAL
A. mellifera (Apidae)	0	2	3	1	1	7
B. terrestris (Apidae)	0	2	3	1	0	6
D. novaeangliae (Halictidae)	0	2	4	2	1	9
C. gigas (Colletidae)	0	1	1	2	1	5
O. bicornis (Megachilidae)	0	3	2	3	0	8
M. rotundata (Megachilidae)	2	3	0	3	0	8
M. centuncularis (Megachilidae)	1	3	0	4	0	8
M. lapponica (Megachilidae)	1	3	0	2	0	6
M. leachella (Megachilidae)	1	3	0	3	0	7
M. willughbiella (Megachilidae)	3	3	0	2	0	8

Table S4: P450 content in pmol ml^{-1} protein determined by CO-difference spectra and total protein content mg ml^{-1} determined by Bradfords protein assay.

Name	Size in bp	450 nm peak	Amount of P450 nMol ml^{-1}	Amount of protein mg ml^{-1}
C. florisomne CYP9BU3	1566	Y	1.5604	36.35
D.cincta CYP9BU4	1524	Y	1.5824	32.86
H. truncorum CYP9BU5	1581	N	N/A	29.68
H. truncorum CYP9BU6	1554	N	N/A	19.27
O. lignaria CYP9BU1	1566	Y	5.6484	36.61
O. bicornis CYP9BU1	1554	Y	4.5495	31.96
O. bicornis CYP9BU2	1530	Y	1.2308	36.42
M. rotundata CYP9DM1	1497	Y	0.6593	24.69
M. rotundata CYP9DM2	1596	N	N/A	25.87

Table S5: Statistical analysis of parent compound depletion and hydroxy-metabolite production [TCP-OH and IMI-OH] after incubation of cytochrome P450s with two neonicotinoid insecticides [thiacloprid (TCP) and imidacloprid (IMI)]. Welch's t-tests were used to compare insecticide depletion in the presence and absence of NADPH and the level of TCP-OH versus IMI-OH production in the presence of NADPH.

**Enzyme**	**Mean depletion TCP** (± SEM)	**p value: TCP NADPH- vs TCP NADPH+**	**Mean depletion IMI** (± SEM)	**p value: IMI NADPH- vs IMI NADPH+**	**p value: TCP-OH vs IMI-OH**
C. florisomne CYP9BU3	360.369 (±60.48)	0.0050**	345.549 (±33.96)	0.0495*	0.0017**
D. cincta CYP9BU4	157.884 (±46.95)	0.0277*	220.538 (±62.36)	0.2742	0.0003***
H. truncorum CYP9BU5	62.279 (±33.84)	0.0711	220.931 (±162.2)	0.3363	0.0022**
O. lignaria CYP9BU1	-70.277 (±136.5)	0.3265	262.274 (±43.21)	0.2168	0.4771
M. rotundata CYP9DM1	-94.98 (± 59.45)	0.2366	-6.648 (± 67.41)	0.9281	N/A (values all <LOQ)
M. rotundata CYP9DM2	-6.550 (±38.64)	0.4373	148.541 (±205.9)	0.2782	0.1419

Table S6: Statistical analysis of hydroxy-metabolite production [TCP-OH and IMI-OH] after incubation of cytochrome P450s with two neonicotinoid insecticides [thiacloprid (TCP) and imidacloprid (IMI)]. One-way ANOVA and *post-hoc* pairwise comparisons (Dunnett's multiple comparisons test) of metabolite production for each P450 compared to that of *M. rotundata* CYP9DM2.

ANOVA summary	LC-MS/MS TCP	LC-MS/MS IMI
F	570.3	5355
p value	<0.0001****	<0.0001****

	Adjusted P Value	
Dunnett's multiple comparisons test	LC-MS/MS TCP	LC-MS/MS IMI
DM2 vs Cf_BU3	<0.0001****	<0.0001****
DM2 vs Dc_BU4	<0.0001****	<0.0001****
DM2 vs Ht_BU5	0.0010**	<0.0001****
DM2 vs Ol-BU1	<0.0001****	<0.0001****

Table S7: Catalytic pocket volume (Å^3) and druggability score for the three-dimensional AlphFold2 computational models of *A. mellifera* CYP9Q3, *M. rotundata* CYP9DM1 and *C. florisomne* CYP9BU3.

P450	Catalytic pocket size (Å^3)	Druggability
A. mellifera CYP9Q3	1407	0.80
C. florisomne CYP9BU3	3043	0.90
M. rotundata CYP9DM1	1529	0.86

Table S8: Topology and binding energies for the most relevant access tunnel for TCP and IMI for *A. mellifera* CYP9Q3, *C. florisomne* CYP9BU3 and *M. rotundata* CYP9DM1. Selection based on energy barrier E_{max} values, activation energy (E_a) and length (Å).

P450	E_{bound} (Kcal/mol)	E_{max} (Kcal/mol)	$E_{surface}$ (Kcal/mol)	E_a (Kcal/mol)	ΔE_{BS} (Kcal/mol)	Length Å	Bottleneck radius Å
				**TCP**			
A_mellifera CYP9Q3	-4.9	-3.1	-3.7	0.6	-1.2	24.6	1.6
M_rotundata CYP9DM1	-5.7	9.4	-1.9	11.3	-3.8	19.3	1.1
C_florisommne CYP9BU3	-6.6	-3.1	-3.4	0.3	-3.2	11.1	1.7
				**IMI**			
**P450**	E_{bound} (Kcal/mol)	E_{max} (Kcal/mol)	$E_{surface}$ (Kcal/mol)	E_a (Kcal/mol)	ΔE_{BS} (Kcal/mol)	Length Å	Bottleneck radius Å
A_mellifera CYP9Q3	-6.0	-4.4	-3.6	-0.8	-2.4	24.6	1.6
M_rotundata CYP9DM1	-6.2	8.5	-2.7	11.2	-3.5	19.3	1.1
C_florisommne CYP9BU3	-6.6	-3.2	-4.0	0.8	-2.6	11.1	1.7

Table S9: Table showing all the access tunnels for *A. mellifera* CYP9Q3, *C. florisomne* CYP9BU3 and *M. rotundata* CYP9DM1.

CYP9Q3	Bottle neck radius Å	Length Å	Distance to surface Å	Curvature	Throughput	No of residues	No of bottlenecks
Tunnel 1	1.5	19.3	13.7	1.4	0.65	29	1
Tunnel 2	1.6	24.6	16.9	1.5	0.60	33	1
Tunnel 3	1.1	21.2	15.7	1.3	0.49	28	1
Tunnel 4	0.9	21.4	13.6	1.6	0.46	30	1
CYPBU3							
Tunnel 1	1.7	11.1	9.2	1.2	0.75	17	1
Tunnel 2	1.2	15.2	11.6	1.3	0.58	25	1
Tunnel 3	1.0	15.0	10.2	1.5	0.57	21	1
Tunnel 4	1.1	25.8	15.4	1.7	0.55	38	1
Tunnel 5	1.0	18.3	11.9	1.5	0.53	26	1
Tunnel 6	1.3	19.6	14.0	1.4	0.52	30	1
CYPDM1							
Tunnel 1	1.1	19.3	14.5	1.3	0.56	32	1
Tunnel 2	1.0	20.0	12.8	1.6	0.36	28	1
Tunnel 3	1.0	25.4	18.1	1.4	0.30	38	1
Tunnel 4	0.9	22.8	15.2	1.5	0.28	32	1

Table S10: Binding energies for *A. mellifera* CYP9Q3, *C. florisomne* CYP9BU3 and *M. rotundata* CYP9DM1with ligands TCP and IMI

CYP9Q3 TCP	E_{bound} Kcal/mol	E_{max} Kcal/mol	$E_{surface}$ Kcal/mol	E_a Kcal/mol	ΔE_{BS} Kcal/mol
Tunnel 1	-4.6	-0.5	-3.1	2.6	-1.5
Tunnel 2	-4.9	-3.1	-3.7	0.6	-1.2
Tunnel 3	-4.6	11.2	-0.4	11.6	-4.2
Tunnel 4	-5.5	3.4	1.7	1.7	-6.2
CYP9Q3 IMI	E_{bound} Kcal/mol	E_{max} Kcal/mol	$E_{surface}$ Kcal/mol	E_a Kcal/mol	ΔE_{BS} Kcal/mol
Tunnel 1	-6.0	-1.5	-4.8	3.3	-1.2
Tunnel 2	-6.0	-4.4	-3.6	-0.8	-2.4
Tunnel 3	-5.7	8.3	-2.0	10.3	-3.7
Tunnel 4	-6.3	1.8	1.3	0.5	-7.6
CYP9BU3 TCP	E_{bound} Kcal/mol	E_{max} Kcal/mol	$E_{surface}$ Kcal/mol	E_a Kcal/mol	ΔE_{BS} Kcal/mol
Tunnel 1	-6.6	-3.1	-3.4	0.3	-3.2
Tunnel 2	-6.1	3.3	-1.8	5.1	-4.3
Tunnel 3	-6.6	0.1	-3.8	3.9	-2.8
Tunnel 4	-5.7	1.6	-4.8	6.4	-0.9
Tunnel 5	-6.6	17.2	-5.7	22.9	-0.9
Tunnel 6	-5.1	9.6	-0.7	10.3	-4.4
CYP9BU3 IMI	E_{bound} Kcal/mol	E_{max} Kcal/mol	$E_{surface}$ Kcal/mol	E_a Kcal/mol	ΔE_{BS} Kcal/mol
Tunnel 1	-6.6	-3.2	-4.0	0.8	-2.6
Tunnel 2	-6.5	1.5	-2.5	4.0	-4.0
Tunnel 3	-6.5	0.5	-4.8	5.3	-1.7
Tunnel 4	-6.4	1.6	-5.9	7.5	-0.5
Tunnel 5	-7.1	14.8	-6.0	20.8	-1.1
Tunnel 6	-6.4	5.2	-1.4	6.6	-5.1
CYP9DM1 TCP	E_{bound} Kcal/mol	E_{max} Kcal/mol	$E_{surface}$ Kcal/mol	E_a Kcal/mol	ΔE_{BS} Kcal/mol
Tunnel 1	-5.7	9.4	-1.9	11.3	-3.8
Tunnel 2	-5.7	22.1	4.5	17.6	-10.2
Tunnel 3	-5.7	21.9	-2.4	24.3	-3.3
Tunnel 4	-6.0	30.2	12.3	17.9	-18.3
CYP9DM1 IMI	E_{bound} Kcal/mol	E_{max} Kcal/mol	$E_{surface}$ Kcal/mol	E_a Kcal/mol	ΔE_{BS} Kcal/mol
Tunnel 1	-6.2	8.5	-2.7	11.2	-3.5
Tunnel 2	-5.5	18.0	4.0	14.0	-9.5
Tunnel 3	-5.5	19.3	-2.9	22.2	-2.6
Tunnel 4	-6.4	30.0	7.5	22.5	-13.9

Table S11: Acute contact LD_{50} (48h) toxicity data for *M. rotundata* and *A. mellifera*

Compound	*Megachile rotundata* LD_{50} 48h (µg ai bee^{-1})	*Apis mellifera* LD_{50} 48h (µg ai bee^{-1})	Fold difference (*A. mellifera* vs *M. rotundata*)
**Neonicotinoids**			
**Thiacloprid**	0.015[a]	38.82[c]	2588
**Imidacloprid**	0.001[a]	0.081[c]	81
**Acetamiprid**	0.179[b]	8.09[c]	45
**Butenolides**			
**Flupyradifurone**	0.092[a f]	>100[e]	1087
**Pyrethroids**			
***Tau*- fluvalinate**	0.061[b]	12.0[c]	196
**Deltamethrin**	0.004[b]	0.0015[c]	-0.375
**Organophosphates**			
**Coumaphos**	0.557[b]	20.29[d]	36
**Chlorpyrifos**	0.017[b]	0.07[d]	4.1

[a] Hayward et al., 2019
[b] Hayward, 2021
[c] Lewis et al., 2016
[d] Sanchez-Bayo & Goka, 2014
[e] Nauen et al., 2015
[f] LD_{50} 72h
